# New depsidones from *Garcinia parvifolia* and their antidiabetic, anti-tyrosinase, and antioxidant activities

**DOI:** 10.1039/d6ra06497c

**Published:** 2026-10-22

**Authors:** Rico Ramadhan, Azeana Zahari, Irmayanti Tri Kurnia, Anh-Khoa Nguyen, Sehanat Prasongsuk, Yazdaniyar Fajri Halimi Firdaus, Thanyada Rungrotmongkol, Preecha Phuwapraisirisan, Dinda Ayu Lestari, Jirapast Sichaem, Kautsar Ul-Haq, Aziz Ahmad Bukhori, Devi Nawang Ariningtyas, Lukman Riyadi

**Affiliations:** a Division of Exploration and Synthesis of Bioactive Compounds (ESBC), University CoE – Research Center for Bio-Molecule Engineering (BIOME), Universitas Airlangga Surabaya 60115 Indonesia; b Department of Chemistry, Faculty of Science and Technology, Universitas Airlangga Surabaya 60115 Indonesia rico.ramadhan@fst.unair.ac.id; c Plant Biomass Utilization Research Unit, Department of Botany, Faculty of Science, Chulalongkorn University Bangkok 10330 Thailand; d Department of Chemistry, Faculty of Science, Universiti Malaya Kuala Lumpur 50603 Malaysia; e Faculty of Pharmacy, Ton Duc Thang University Ho Chi Minh City Vietnam; f Center of Excellence in Biocatalyst and Sustainable Biotechnology, Department of Biochemistry, Faculty of Science, Chulalongkorn University Bangkok 10330 Thailand; g Program in Bioinformatics and Computational Biology, College of Interdisciplinary and Integrative Studies, Chulalongkorn University Bangkok 10330 Thailand; h Center of Excellent in Natural Products (CENP), Department of Chemistry, Faculty of Science, Chulalongkorn University Bangkok 10330 Thailand; i Department of Chemistry, Faculty of Science and Technology, Thammasat University Lampang Campus Lampang 52190 Thailand; j Environmental Office of Balikpapan Balikpapan 76115 East Kalimantan Indonesia

## Abstract

In the search for potent bioactive compounds derived from medicinal plants, *Garcinia parvifolia* from East Kalimantan has demonstrated significant bioactivity consistent with established scientific criteria. Bioassay-guided isolation of the fruit extract resulted in two new depsidones named methyl garcidepsidone B (1) and parvifoliricodone A (6), two known analogues, a phloroglucinol, and a furfural derivative. Their structures were determined by spectroscopic techniques. The isolated compounds were subjected to assessments for their potential antidiabetic effects through an α-glucosidase inhibition assay, anti-tyrosinase activity, and antioxidant properties. Out of the isolated compounds, methyl garcidepsidone B (1) was the most potent inhibitor against α-glucosidase compared with acarbose as a positive standard. In the antioxidant assay, all isolated compounds showed significant free radical scavenging activity against ABTS, with the exception of compound 4. Regarding tyrosinase inhibitory activity, all isolated compounds exhibited low enzyme inhibition activity. Additionally, *in silico* molecular docking studies showed that compounds 1–3 exhibited favorable conformation and binding affinities within the enzymatic pockets of α-glucosidase. These findings indicate that the indigenous *G. parvifolia* fruit may serve as a natural source of antidiabetic, antioxidant, and anti-tyrosinase compounds, with potential for further pharmacological investigation.

## Abstract 2

In the search for potent bioactive compounds derived from medicinal plants, *Garcinia parvifolia* from East Kalimantan has demonstrated significant bioactivity consistent with established scientific criteria.

## Graphical abstract

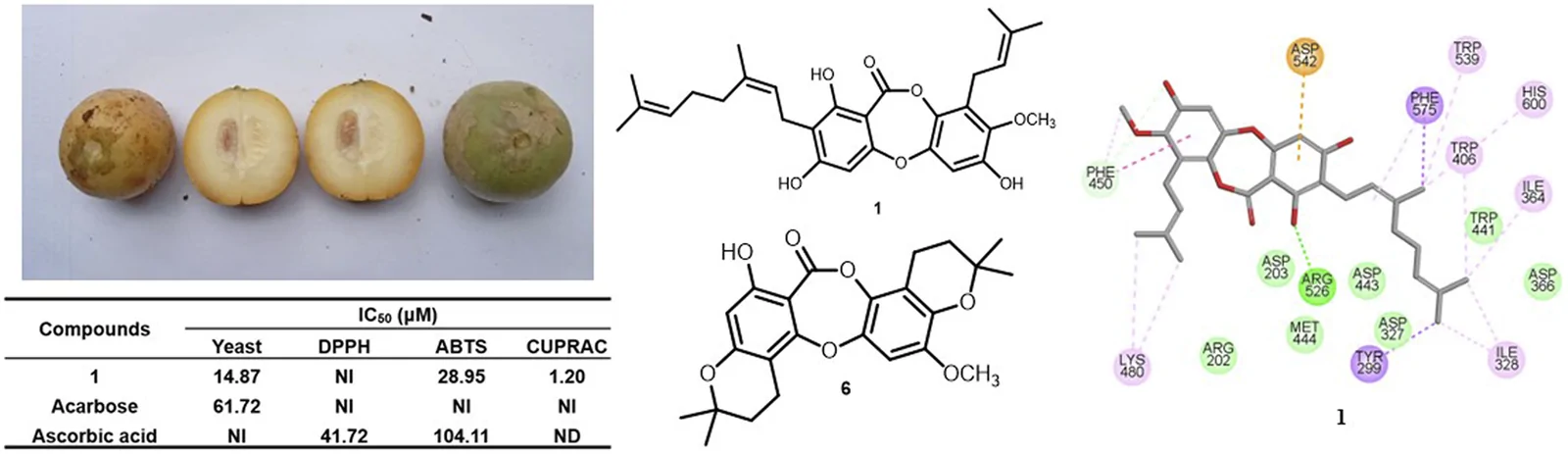

## Introduction

*Garcinia parvifolia* (Clusiaceae) a lowland-forest species whose natural range extends across Borneo, including East Kalimantan has been shown to accumulate bioactive xanthones such as parvixanthone A and rubraxanthone alongside phloroglucinol and depsidone constituents.^1^ To date, however, the phytochemical characterisation of this species fruits has drawn almost exclusively on material collected in Peninsular Malaysia and Thailand, while the populations native to East Kalimantan remains essentially uninvestigated. This plant family consists of over 1000 species primarily found in tropical regions. *Garcinia*, a genus within the *Guttiferae* (Clusiaceae) family, consists of numerous polygamous trees or shrubs and is distributed in tropical Asia, including the East Kalimantan region. Members of this genus are well recognized for their prolific biosynthesis of structurally diverse secondary metabolites,^2^ including xanthones,^3^ benzophenones,^4,5^ depsidones,^6,7^ bioflavonoids,^8^ biphenyl,^9^ and triterpenoids.^10^ These secondary metabolites exhibit a broad spectrum of biological and pharmacological activities, including antioxidant,^11^ α-glucosidase inhibition,^12^ anti-tyrosinase,^13^ anti-HIV,^14^ anti-inflammatory,^15^ anti-cancer,^16^ antibacterial,^17^ and anti-Alzheimer properties.^18^

In this recent study, phytochemicals in plants from East Kalimantan are used as potential sources of bioactive compounds, while novel depsidones have been identified in the fruits of *G. parvifolia* cultivated in this region, exhibiting antidiabetic, anti-tyrosinase, and antioxidant activities. Among these compounds, two novel depsidones (designated as 1 and 6) featuring the dibenzo[*b*,*e*][1,4]-dioxepin-11-one structure, along with other known compounds (2–5), were identified in the dichloromethane-soluble fraction. This discovery represents the first reported instance of a higher plant producing significant quantities of depsidones, which were previously regarded as compounds predominantly associated with lichens.^19^ The biological activities of these bioactive compounds (1–6) were assessed through assays measuring α-glucosidase and tyrosinase inhibitory effects, as well as antioxidant properties and *in silico* study.

## Experimental

### Materials

The fresh fruits of *G. parvifolia* were collected from Balikpapan Botanical Garden and identified by Mr Trisno, a botanist at Balikpapan Botanical Garden, East Kalimantan. The voucher specimen (ESBC-108-BIOME2023) was deposited at the Research Group of Exploration and Synthesis of Bioactive Compounds (ESBC), University CoE-Research Center for Bio-Molecule Engineering (BIOME), Universitas Airlangga, Indonesia.

### Procedure

#### Extraction and isolation

The dried fruits of *G. parvifolia* (1 kg) were successively and exhaustively extracted with methanol (1 L × 3). Evaporation *in vacuo* reduced the extract to a residue of 100 g. This residue was suspended in aqueous methanol (10%) and re-extracted with *n*-hexane (500 mL × 3), dichloromethane (500 mL × 3), and ethyl acetate (500 mL × 3) to give 10, 35, and 20 g residues, respectively, after concentration *in vacuo*. The dichloromethane soluble fraction was chromatographed by vacuum column chromatography (silica gel 60G Merck) column (120 mm ID × 7 cm length) and eluted with six mobile phases of hexane : EtOAc (1 : 0–0 : 1). The fraction B was chromatographed on Sephadex LH-20 CC using methanol followed by silica gel column chromatography to obtained methyl garcidepsidone B (1, 50 mg) and parvifoliol F (2, 40 mg). The fraction C, upon purification with Sephadex LH-20 CC, yielded subfractions (C1–C4). Subfraction C1 was further purified by repeated Sephadex LH-20 CC to afford garcinisidone B (3, 40 mg). Subfraction C3 was purified by silica gel CC with a solvent system of *n*-hexane : ethyl acetate (3 : 2) to yield 5-acetoxy methyl furfural (4, 35 mg). Fraction D was purified by Sephadex LH-20 CC to yield 4 subfractions (D1–D4). Subfraction D2 was further purified by Sephadex LH-20 CC with a solvent system of dichloromethane : methanol (4 : 2) to yield garcinisidone A (5, 50 mg) and parvifoliricodone A (6, 46.6 mg).

Methyl garcidepsidone B (1): white powder; UV *λ*_max_ (MeOH) (log *ε*) 206 (4.46), 282 (3.89); IR (KBr) *ν*_max_ 3346, 2965, 2913, 2853,1649, 1622 cm^−1^; ^1^H NMR (CDCl_3_, 400 MHz) and ^13^C NMR (CDCl_3_, 100 MHz) spectroscopic data, as shown in Table S1; HRMS *m*/*z* 517.2199 [M + Na]^+^ (calcd for C_29_H_35_NaO_7_ 517.2202).

Parvifoliol F (2): yellow gum; ^1^H NMR (CDCl_3_, 400 MHz) and ^13^C NMR (CDCl_3_, 100 MHz) spectroscopic data, see SI. The ^1^H and ^13^C NMR spectral data were in good agreement with those of previous studies.^20^

Garcinisidone B (3): yellow powder; ^1^H NMR (CDCl_3_, 400 MHz) and ^13^C NMR (CDCl_3_, 100 MHz) spectroscopic data, see SI. The ^1^H and ^13^C NMR spectral data were in good agreement with those of previous studies.^21^

5-Acetoxy methyl furfural (4): yellow gum; ^1^H NMR (CDCl_3_, 400 MHz) and ^13^C NMR (CDCl_3_, 100 MHz) spectroscopic data, see SI. The ^1^H and ^13^C NMR spectral data were in good agreement with those of previous studies.^22^

Garcinisidone A (5): white powder; ^1^H NMR (acetone-d_6_, 400 MHz) and ^13^C-APT NMR (acetone-d_6_, 100 MHz) spectroscopic data, see SI. The ^1^H and ^13^C NMR spectral data were in good agreement with those of previous studies.^23^

Parvifoliricodone A (6): yellow powder; UV *λ*_max_ (MeOH) (log *ε*) 206 (4.16), 282 (3.83); IR (KBr) *ν*_max_ 3499, 3337, 2974, 1626, 1459 cm^−1^; ^1^H NMR (CDCl_3_, 400 MHz) and ^13^C NMR (CDCl_3_, 100 MHz) spectroscopic data, as shown in Table 1; HRMS *m*/*z* 427.1761 [M + H]^+^ (calcd for C_24_H_26_O_7_^+^ 427.1757).

**Table 1:** ^1^H (400 MHz) and ^13^C NMR (150 MHz) data of 1 and 6 in CDCl_3_ (*δ* in ppm, *J* in Hz)

No.	1		No.	6	
	*δ*_H_ (H, mult., *J* = Hz)	*δ*_C_		*δ*_H_ (H, mult., *J* = Hz)	*δ*_C_
1	11.31 (1H, s)	162.6	1	11.35 (1H, s)	159.3
2		111.1	2	6.23 (1H, s)	101.4
3	6.35 (1H, brs, OH)	162.4	3		163.1
4	6.28 (1H, s)	100.5	4		106.2
4a		159.8	4a		161.6
5a		146.9	5a		146.8
6	6.71 (1H, s)	105.5	6	6.69 (1H, s)	105.7
7		147.0	7		142.7
8		142.6	8		147.3
9		120.8	9		129.2
9a		133.3	9a		136.2
11		168.6	11		169.2
11a		98.7	11a		97.2
12	3.40 (2H, d, 7.32)	22.1	12		76.7
13	5.20 (1H, m)	123.7	13	1.77 (2H, m)	31.1
14		139.9	14	2.61 (2H, t, 6.72)	16.5
15	2.06 (2H, m)	39.7	15	1.32 (3H, m)	26.7
16	2.06 (2H, m)	26.3	16	1.32 (3H, m)	26.7
17	5.03 (1H, m)	128.3	17		71.1
18		132.3	18	1.77 (2H, m)	44.0
19	1.58 (3H, s)	17.8	19	2.87 (2H, m)	19.7
20	1.66 (3H, m)	25.7	20	1.31 (3H, m)	29.1
21	1.79 (3H, m)	16.3	21	1.31 (3H, m)	29.1
22	3.47 (2H, d, 6.72)	24.2	7-OCH_3_	3.76 (3H, s)	62.1
23	5.21 (1H, m)	121.3			
24		136.3			
25	1.81 (3H, m)	18.1			
26	1.67 (3H, m)	25.8			
8-OCH_3_	3.76 (3H, s)	61.9			

### α-Glucosidase inhibitory activity

The α-glucosidase inhibitory activity of isolated compounds was examined using the chromogenic method described by a previous study with slight modification.^24^ Briefly, various concentrations (6.25–500 µg mL^−1^, initial concentrations) of isolated compounds (10 µL) were added with a phosphate buffer with pH 6.8 (50 µL), followed by 40 µL (0.4 U of α-glucosidase from *Saccharomyces cerevisiae* type I), lyophilized powder using *p*-nitrophenyl α-d-glucoside as substrate. After pre-incubation at 37 °C for 10 minutes, 40 µL of *p*-nitrophenyl glucoside (10 mM) was added. Then, 100 µL of 0.1 M Na_2_CO_3_ solution was added, and after 30 minutes of reaction at 37 °C, the absorbance was recorded at 405 nm. Each sample was performed and measured in technical triplicate, and the data is reported as the mean one standard deviation (SD). Acarbose was assayed under identical conditions as positive control, and the IC_50_ values (the sample concentration necessary to inhibit 50% of enzyme) were obtained by extrapolating the regression analysis results. Assays were performed in technical replicates (*n* = 3), and are expressed as mean ± SD.

### Tyrosinase inhibitory activity

The *in vitro* anti-tyrosinase activity was determined as previously reported with minor modification.^25^ Five different concentrations of the respective test sample dissolved in dimethyl sulfoxide were prepared (6.25–500 µg mL^−1^). The reaction mixture contained 120 µL of 2.5 mM l-DOPA in 80 mM phosphate buffer (PB), pH 6.8, 30 µL of PB, and 10 µL of the sample solution in DMSO. The mixture was mixed and pre-incubated at 25 °C for 10 minutes, before 40 µL of mushroom tyrosinase in 80 mM PB was added and incubated at 25 °C for 5 minutes. The absorbance was then measured at 475 nm by a microplate reader (Multiskan SkyHigh, Thermo Scientific). In parallel, kojic acid was used as a positive reference standard of a diphenolase inhibitor. Each sample was performed and measured in technical triplicate (*n* = 3), and the data is reported as the mean one standard deviation (SD).

### DPPH free radicals scavenging activity

The DPPH (2,2-diphenyl-1-picrylhydrazyl) free radical was used to analyze the isolated compounds free radical scavenging potential. The method described in the previous study with minor modifications.^26^ Briefly, 20 µL of isolated compounds with various concentrations (6.25–500 µg mL^−1^) were mixed with 0.1 mM of DPPH methanol solution. The reaction mixture was incubated in the dark, and after 30 minutes, absorbance was measured on a 96-well Thermo Scientific Multiskan SkyHigh RE 6.1.1 microplate reader at 517 nm using a methanol blank as a reference. The percentage of free radical scavenging activity was calculated using the eqn (1) as follows:

1 % scavenging activity = (Abs_control_ − Abs_sample_)/Abs_control_ × 100.

The results were expressed as IC_50_ values (the sample concentration required to inhibit 50% of DPPH radicals). This value was determined by extrapolating the regression analysis.

### ABTS˙^+^ free radicals scavenging activity

The antiradical activity was examined using the ABTS˙^+^ free radical decolorization assay developed in a previous study.^26^ In brief, 20 µL of isolated samples with various concentrations was mixed with ABTS˙^+^ working solution in a 96-well microplate. The absorption was measured spectrophotometrically at 750 nm after 30 minutes, and the scavenging activity was calculated using eqn (1). The IC_50_ values (the sample concentration necessary to inhibit 50% of free radical) were obtained by extrapolating the regression analysis results.

### Cupric reducing antioxidant activity (CUPRAC)

The isolated compounds from the fruits of *G. parvifolia* were determined using the CUPRAC (Cupric ion Reducing Antioxidant Capacity) assay, performed with the previous method used with slight modification.^27^ During the experiment, 100 µL of isolated compounds with various concentrations was added with 50 µL of CuCl_2_ solution, 50 µL of NH_4_Ac buffer, and 50 µL of neocuproine. After 30 minutes of incubation, the reaction mixture was measured spectrophotometrically at 450 nm. The results were interpreted by calculating the mmol g^−1^ (standard curve; see SI) Trolox Equivalent Antioxidant Capacity (TEAC) compared to the standard antioxidant substance Trolox, which has a high reduction potential.

### Ferric reducing antioxidant power (FRAP)

The efficacy of isolated compounds from the fruits of *G. parvifolia* in reducing iron was assessed using a previous method with a slight modification.^28^ This assay was conducted based on the reduction of a colorless iron complex of Fe^3+^-tripyridyltriazine to a blue complex of Fe^2+^-tripyridyltriazine. The FRAP reagent was combined with a freshly prepared 300 mM acetate buffer, 40 mM HCl, 10 mM TPTZ, and 20 mM FeCl_3_·6H_2_O. The standard curves (see SI) were created using various concentrations of FeSO_4_·7H_2_O, and all solutions were prepared under fresh conditions. To conduct the assay, 20 µL of the sample solution with various concentrations, 80 µL of distilled water, and the FRAP reagent were mixed and incubated at 37 °C for 30 minutes. The absorbance was then measured at 593 nm, and the difference between the absorbance of the samples and the absorbance of the blank was calculated and used to obtain the FRAP value and the reduction capacity of the isolated compounds.

### Molecular docking analysis

The molecular docking was performed using the GOLD software to investigate protein–ligand interactions, according to the standard protocol.^29^ The crystal structure of N-terminal human maltase-glucoamylase (Nt-MGAM) was selected as a representative model for α-glucosidase (PDB ID: 3L4W).^30^ The three-dimensional structures of the isolated compounds were constructed using the pKaNET-Cloud tool at pH 7.4.^31^ The co-crystallized ligands and protein structures were prepared using the GOLD preparation tool, which was used to add hydrogen atoms to the ligands and amino acid residues and remove water molecules. While co-solvent was removed by using Discovery Studio. Docking calculations were conducted within a 10 Å radius sphere centered on the binding pocket of the miglitol. For each ligand, 100 docking poses were generated and evaluated using the GOLD fitness scoring function. The highest-scoring pose was selected for further analysis and visualized using Discovery Studio 2024 and ChimeraX 11.1.^32^ The reliability of the docking protocol was confirmed through re-docking of the co-crystallized ligand into the Nt-MGAM active site, which successfully reproduced the experimentally observed binding pose with good agreement (see SI).

### Statistical analysis

The obtained quantitative data were subjected to descriptive analysis. All measurements were conducted in technical triplicates. The quantitative data were tabulated and exported into SigmaPlot 12.5 for further analysis, which was carried out descriptively.

## Results and discussion

The methanol extract from the fruit of *G. parvifolia* was subjected to liquid–liquid partition to obtain hexane, dichloromethane, and ethyl acetate fractions. The dichloromethane-soluble fraction was loaded onto a Sephadex LH-20 column and further separated using various chromatographic methods to afford four derivative depsidones, one phloroglucinol derivative, and one furan derivative, including two undescribed structures (1 and 6). The structures elucidation of the previously reported derivatives was determined after comparison of their NMR spectroscopic data in the literature, including parvifoliol F (2), garcinisidone B (3), 5-acetoxy methyl furfural (4), and garcinisidone A (5).

Methyl garcidepsidone B (1) was obtained as a yellow colorless oil and showed a molecular formula to be C_29_H_35_NaO_7_^+^ based on an [M + Na]^+^ ion at *m*/*z* 517.2199 in the HRMS data. It exhibited UV and IR absorption bands similar to those of garcidepsidone B. The ^1^H NMR and HSQC spectra revealed two aromatic methines (*δ*_H_ 6.28 and 6.71). The ^13^C NMR, in accordance with the HSQC spectrum, exhibited one ester (*δ*_C_ 162.6), two methine (*δ*_C_ 100.5 and 105.5), one methoxy (*δ*_C_ 61.9), and seven oxygenated aromatic carbons (*δ*_C_ 162.6, 162.4, 159.8, 146.9, 147.0, 142.6, and 133.3). The spectroscopic data suggested that 1 was a depsidone with three member rings. The ^1^H NMR (Table 1), which showed one set of signals corresponding to prenyl group [*δ*_H_ 1.67 (m, 1H), *δ*_H_ 1.81 (3H, m), *δ*_H_ 3.47 (2H, d, 6.72), and *δ*_H_ 5.21 (1H, m)], one geranylated group was confirmed by the multiplets at *δ*_H_ 2.06 (4H, m) corresponding to two adjacent CH_2_ groups. The ^1^H and ^13^C NMR data in combination with the results of HMQC indicated that 1 had five methyl groups of isoprenyl origin. The presence of geranylated group at C-2 was confirmed by the correlation of H-12 (*δ*_H_ 3.40) with C-2 (*δ*_C_ 111.1) and C-14 (*δ*_C_ 139.9). Similarly, prenylated group was located at C-9 as the correlation evidence of H-22 (*δ*_H_ 3.47) with C-9a (*δ*_C_ 133.3) and C-24 (*δ*_C_ 139.3). The presence of a methoxy group at C-8 was also observed with the correlation of *δ*_H_ 3.76 and C-8 (*δ*_C_ 142.6). With careful investigation, the ^1^H, ^13^C NMR (Table 1), HMQC, and HMBC data were similar to those of garsidepsidone B except for the replacement of hydroxyl on C-8 with a methoxy group.^19^ The selected HMBC correlation of compound 1 was shown by arrows in Fig. 2. Therefore, the structure of compound 1 was determined as methyl garcidepsidone B (Fig. 1).

**Fig. 1:**
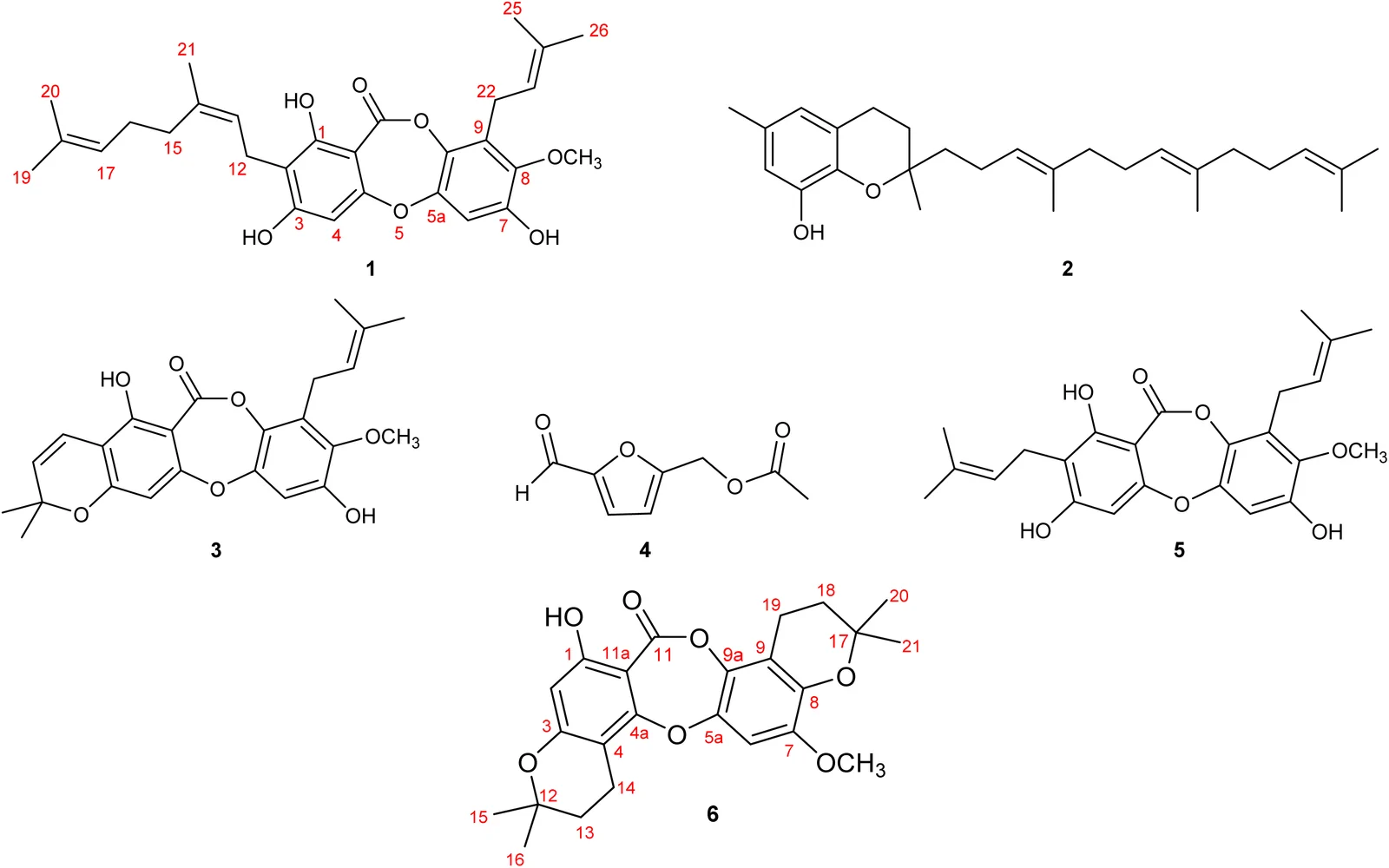
Isolated compounds (1–6) from the fruits of *Garcinia parvifolia*.

Parvifoliricodone A (6) was obtained as a white powder and showed a molecular ion at *m*/*z* 427.1761 [M + H]^+^ (calcd for C_24_H_26_O_7_ 427.1757) in its HRMS. Analysis of the ^1^H NMR and HSQC spectra of parvifoliricodone A (6) identified two aromatic methine signals at *δ*_H_ 6.23 and 6.69. Its ^13^C NMR spectrum displayed resonances attributable to an ester carbonyl (*δ*_C_ 169.2), two aromatic methines (*δ*_C_ 101.4 and 105.7), a methoxy group (*δ*_C_ 62.1), and seven oxygenated aromatic carbons (*δ*_C_ 136.2, 142.7, 146.8, 147.3, 159.3, 161.6, and 163.1), with the protonated carbons assigned on the basis of the HSQC correlations. The close similarity of these spectroscopic features to those of 1 suggested that 6 contained a related tricyclic depsidone framework which contains two aromatic rings. Two singlet aromatic proton signals were observed at *δ*_H_ 6.69 (H-6) and 6.23 (H-2), a methoxy signal at *δ*_H_ 3.76 (3H, 7-OMe), four benzylic proton signals were observed at *δ*_H_ 2.87 (2H, H-19) and 2.61 (2H, *J* = 6.7 Hz, H-14) coupling to two methylene protons at *δ*_H_ 1.77 (4H, m, H-13 and H-18) and four methyl proton signals at *δ*_H_ 1.32 (6H, m) and 1.31 (6H, m), suggesting the presence of two dimethylchromone rings consistent with a DBE value of 12 for this compound. The methylene at *δ*_H_ 2.61 (H-14) exhibits HMBC correlations with two quaternary carbon signals at *δ*_C_ 163.1 (C-3) and 161.6 (C-4a) reflecting that one of the dihydrochromene rings is attached to positions C-3 and C-4. The second dihydrochromene ring was substituted to positions at C-8 and C-9 due to the correlations observed in the HMBC spectrum between the methylene proton signal at *δ*_H_ 2.87 (H-19) and the carbon signals at *δ*_C_ 129.2 (C-9) and 136.2 (C-9a). In the HMBC spectrum, the proton signals due to the hydroxyl group at *δ*_H_ 11.35 (1-OH) indicated key cross-peaks to a quaternary carbon signal at *δ*_C_ 97.2 (C-11a) and a protonated carbon signal at *δ*_C_ 101.4 attributed to C-2. The singlet aromatic proton at *δ*_H_ 6.23 (H-2) was observed to correlate with two carbon signals at *δ*_C_ 163.1 and 159.3 ascribed to C-3 and C-1, respectively. Furthermore, the methylene at *δ*_H_ 2.61 (2H, t, *J* = 6.72, H-14) exhibits HMBC correlations with three quaternary carbon signals at *δ*_C_ 163.1 (C-3), 106.2 (C-4), 161.6 (C-4a), and 31.1 (C-13). Other key cross-peaks were seen in the HMBC spectrum between the singlet aromatic proton signal at *δ*_H_ 6.69 (1H, s, H-6) and *δ*_C_ 146.8 (C-5a), 136.2 (C-9a), 147.3 (C-8), and 142.7 (C-7). The selected HMBC correlation of compound 6 was shown by arrows in Fig. 2. Therefore, the overall structure of compound 6 was determined as parvifoliricodone A (Fig. 1).

**Fig. 2:**
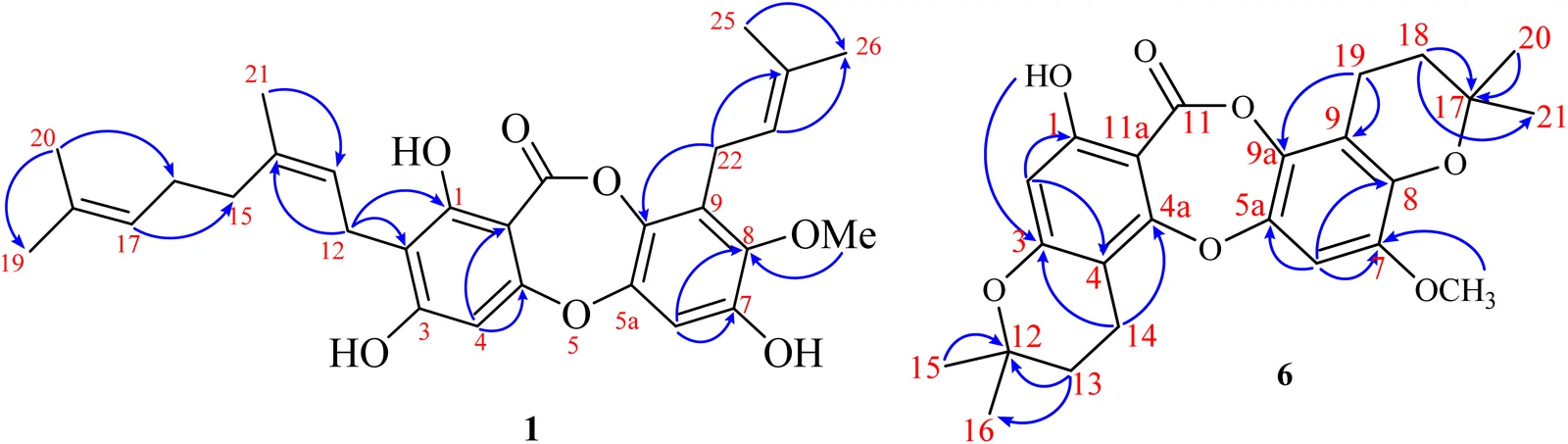
Selected HMBC correlations of compound 1 and 6.

Depsidones are predominantly biosynthesized in lichens *via* the polyketide pathway and typically feature 3,8-dioxygenation as well as methyl substituents at the C-1, C-6, and C-9 positions.^8,33^ However, several prenylated depsidones have been identified within the genus *Garcinia*,^8,33,34^ which is recognized as a prolific source of aromatic compounds derived from the shikimate and mevalonate pathways. It is plausible that the biosynthesis of these prenylated depsidones involves Baeyer–Villiger rearrangements following hydroperoxylation of precursor xanthones, together with the incorporation of prenyl units derived from the mevalonate pathway.^8,35^

Compounds 1–6 were isolated and subsequently assessed for their α-glucosidase inhibitory activity. In addition, their anti-tyrosinase activity was evaluated, and their antioxidant activities were determined using DPPH and ABTS also reducing-capacity by CUPRAC, and FRAP assays. Of tested compounds, 1 and 2 showed strikingly potent activity, compared to all other isolated compounds in the α-glucosidase inhibitory activity assay (Table 2). This finding in line with previous study revealed that plant phenolic, and *Garcinia*-derived xanthones and benzophenones in particular, therefore commonly display IC_50_ values well below that of acarbose against this enzyme. For Instance, xanthones from fruits of *Garcinia schomburgkiana* showed IC_50_ values of 2.91–26.0 µM, all surpassing acarbose with IC_50_ values of 179 µM,^36^ and norathyriol isolated from *Garcinia mckeaniana* inhibited α-glucosidase with IC_50_ value of 0.07 µM much potent than acarbose (IC_50_ 161.0 µM).^37^ The potency of compound 1 is thus consistent with the established behaviour of this compound class. In addition, the anti-tyrosinase activity of all isolated compounds (1–6) was evaluated using two different substrates, l-tyrosine and l-DOPA. At the tested concentration, all isolated compounds exhibited inhibition below 50% compared with the positive control under identical condition. These values indicate that all isolated compounds possesses only weak tyrosinase inhibitory activity.

**Table 2:** α-Glucosidase and tyrosinase inhibitory activities of 1–6 from *G. parvifolia* fruits

Compounds	IC_50_ (µM) ± SD	Percent inhibition (PI)	
	α-Glucosidase	Tyrosinase^a^	
		l-Tyrosine	l-DOPA
1	14.87 ± 1.64	6.05 ± 0.34	1.94 ± 0.81
2	18.97 ± 1.71	2.72 ± 1.49	2.32 ± 0.20
3	25.61 ± 1.93	31.88 ± 1.28	6.49 ± 0.99
4	NI^b^	5.65 ± 0.31	7.61 ± 0.95
5	21.15 ± 1.26	27.21 ± 0.20	20.19 ± 1.28
6	26.93 ± 2.17	17.68 ± 1.91	10.05 ± 0.45
Acarbose	61.72 ± 0.05	ND	ND
Kojic acid	ND^c^	78.49 ± 0.12	85.94 ± 0.85

a Percentage inhibition (PI) of isolated compounds.

b NI, no inhibition, inhibition effect less than 40%.

c ND, not determined.

To further advance this study, antioxidant activities of all isolated compounds (1–6) were also evaluated (Table 3). Among the isolated compounds, compound 2 exhibited antioxidant activity in the DPPH radical scavenging assay. In contrast, in the ABTS assay, nearly all compounds demonstrated measurable inhibitory effects. Notably, compounds 1 and 5 displayed strong ABTS radical scavenging activity, exceeding that of the positive control standards. Depsidones class have previously been isolated from *Garcinia* genus, paucinervin G from *Garcinia paucinervis* showing notable DPPH and ABTS scavenging activity.^38^ Antioxidant capacity is frequently associated with the modulation of enzymatic browning, since oxidative pathways contribute to melanin formation; recent work on *Calamintha incana* (Sm.) Helder, for example, documented strong antioxidant and antidiabetic activity in another plant species.^39^ According these results, we hypothesize that DPPH's lipophilic nature requires organic solvents where depsidones dissolve poorly, whereas ABTS is soluble in both aqueous and organic media, allowing depsidones to fully express their antioxidant potential. Due to their polyphenolic structures and effective radical scavenging in aqueous media, depsidones show more reliable antioxidant activity in the ABTS assay compared to DPPH.^40^

**Table 3:** Antioxidant and reducing capacities assays of 1–6 from *G. parvifolia* fruits

Compounds	Antioxidant		Reducing capacities	
	SC_50_ (µM) ± SD		CUPRAC TEAC (mmol g^−1^)	FRAP (mmol_Fe_ g^−1^)
	DPPH	ABTS˙^+^		
1	NI^a^	28.95 ± 1.96	1.20 ± 0.02	1.23 ± 0.02
2	73.99 ± 0.53	98.94 ± 2.77	1.64 ± 0.26	1.83 ± 0.02
3	NI	48.41 ± 1.73	2.00 ± 0.08	0.51 ± 0.01
4	NI	NI	0.53 ± 0.01	0.84 ± 0.01
5	NI	17.24 ± 0.50	4.21 ± 0.14	1.40 ± 0.01
6	NI	60.21 ± 1.31	1.33 ± 0.17	0.80 ± 0.01
Ascorbic acid	41.72 ± 1.20	104.11 ± 2.08		
α-Tocopherol	30.67 ± 0.00	51.07 ± 1.47		
Quercetin	23.41 ± 0.00	23.10 ± 0.00		
BHA^b^	46.86 ± 1.17	52.96 ± 0.96		
BHT^c^	78.22 ± 1.92	36.67 ± 0.96		
Trolox	24.97 ± 0.00	55.70 ± 0.84		

a NI: no inhibition, inhibitory effects were less than 40%.

b BHA: butylated hydroxyanisole.

c BHT: butylated hydroxytoluene.

Furthermore, the evaluation of antioxidant activity through electron donation mechanisms specifically focuses on the reduction of Cu^2+^ and ferric ions was measured for all isolated compounds. Among the isolated compounds, compound 5 exhibited the highest CUPRAC and FRAP values, indicating superior reducing antioxidant power. Enhanced activity is attributed to the high concentration of phenolic compounds in compound 5, which act as effective electron donors. This study highlights that the reducing properties of polyphenols, together with their capacity to form stable complexes with transition metals such as iron and copper, play a crucial role in modulating biologically relevant processes involving metal redox states. Additionally, the interaction between copper ions and polyphenols is proposed as a key mechanism driving the antioxidant effects of these natural substances.^41^

Given that the isolated compounds exhibited α-glucosidase inhibition, a molecular docking analysis was also performed for all compounds. The docking should be regarded as a hypothesis-generating analysis as GOLD fitness scores. To confirm the docking procedure was reliable, the co-crystallized ligand was redocked into the defined binding site. Redocking was considered successful when the root means square deviation (RMSD) fell < 2 Å.

The prediction of binding affinities of the isolated compounds within the catalytic site of Nt-MGAM were evaluated using molecular docking analysis (Table 4). Among the compounds tested, compound 1 achieved the highest GOLD fitness score of 67.20. These results suggest that compounds 1 exhibit the most favorable predicted binding affinities among the molecules tested. However, all isolated compounds showed lower docking scores compared to the reference inhibitor acarbose, which attained a score of 81.81. The docking posses suggest that compound 1 may interact with key residue of amino acids offering a possible structural basis for the observed activity that warrants further experimental validation. Furthermore, the binding modes of four compounds exhibiting significant inhibitory activity, consistent with the docking results, are illustrated in Fig. 3. Compounds 1, 3, and 5, which belong to the depsidone class, showed similar interaction patterns within the catalytic pocket. All three compounds interacted with residues Y299 and W406. Compounds 1 and 3 formed hydrogen bonds with R526, while compounds 3 and 5 also established hydrogen bonds with W406 and exhibited π–sulfur interactions with M444. In contrast, compound 2, characterized by a chroman scaffold with a long hydrophobic chain, was predominantly stabilized by hydrophobic interactions with residues Y299, I328, W406, W441, M444, F450, L473, K480, W539, F575, and H600. For comparison, the reference inhibitor acarbose was mainly stabilized by hydrogen bond interactions involving residues D203, T205, D327, M444, R526, D542, and H600. These observations suggest that, in contrast to acarbose, which predominantly engages in polar interactions, the isolated compounds – particularly compound 2 are primarily stabilized within the catalytic pocket through hydrophobic interactions, and the detailed interaction profiles are shown in Fig. 4. The docking results showed a similar trend to that observed in the enzymatic inhibition data. Compound 1 exhibited the strongest inhibitory activity, with an IC_50_ value of 14.87 µM, correlating well with its highest docking score (67.20), suggesting favorable binding within the catalytic site. Similarly, compounds 2, 3, and 5 showed moderate inhibitory activities, with IC_50_ values of 18.97, 25.61, and 21.15 µM, respectively, which correspond to their slightly lower docking scores. In contrast, compound 4, which showed the lowest docking score, exhibited no inhibitory activity, indicating poor binding within the enzyme active site. Move over, to assess concordance between predicted binding and experimental potency, GOLD scores were correlated with pIC_50_ values for the five compounds with measurable α-glucosidase inhibition, except compound 4 was excluded as inactive (see SI). Rank agreement was moderate to strong by Spearman's *ρ* values of 0.70, with a comparable Pearson coefficient (*r* = 0.64, *r*^2^ = 0.41); neither reached significance given the small sample (*n* = 5, *p* > 0.05). Consistent with a ranking rather than affinity predicting role for the scoring function, the lowest scored compound 4 was also experimentally inactive.

**Table 4:** Molecular docking results for isolated compounds (1–6) against Nt-MGAM (PDB ID: 3L4W), reported as GOLD fitness scores

Compounds	GOLD fitness score
1	67.20
2	63.03
3	65.15
4	36.61
5	57.75
6	52.42
Acarbose	81.81

**Fig. 3:**
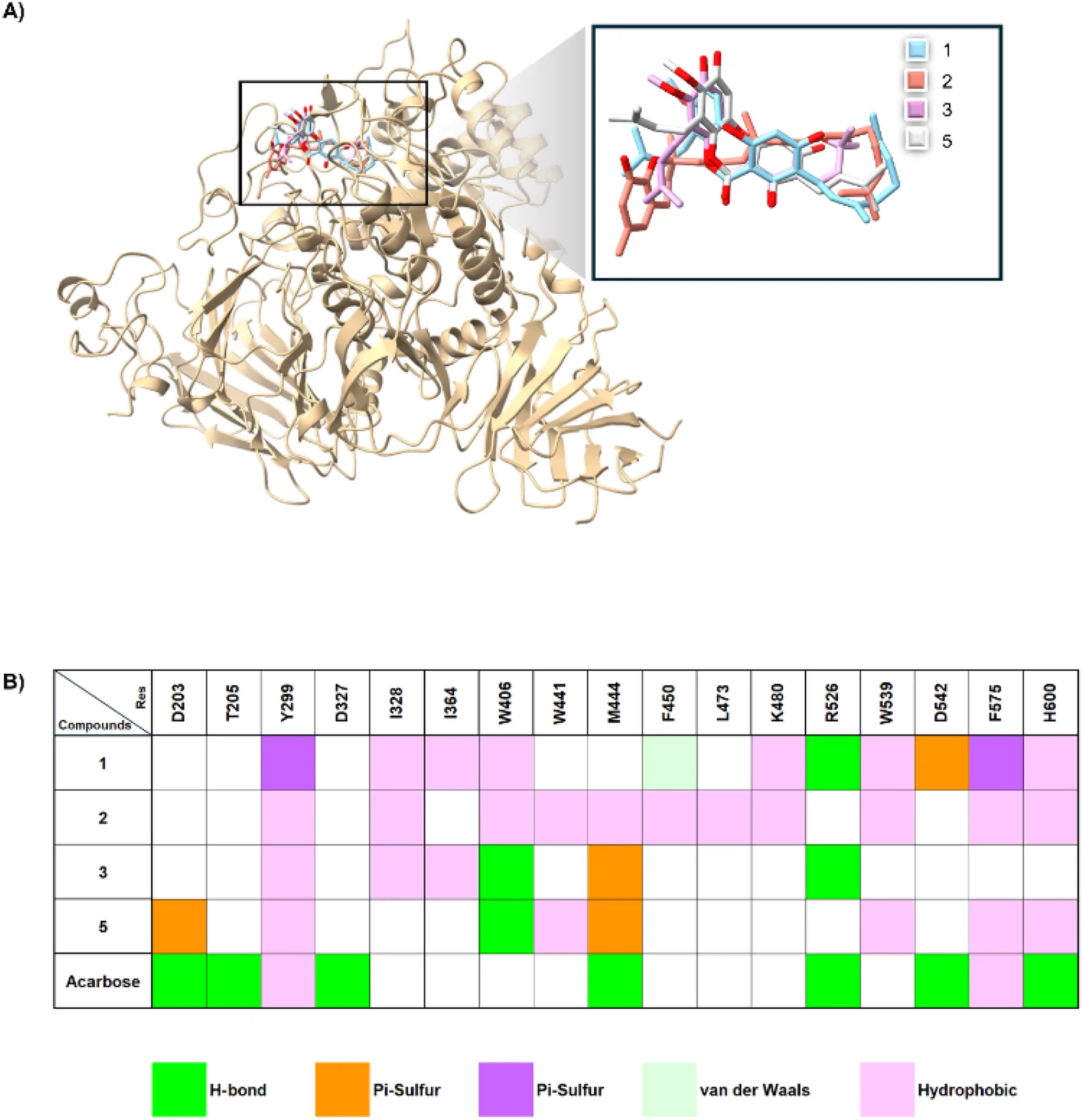
Structural binding analysis of docked ligands against Nt-MGAM. (A) 3D representation of the protein–ligand complexes. (B) Interaction heat map summarizing molecular contacts.

**Fig. 4:**
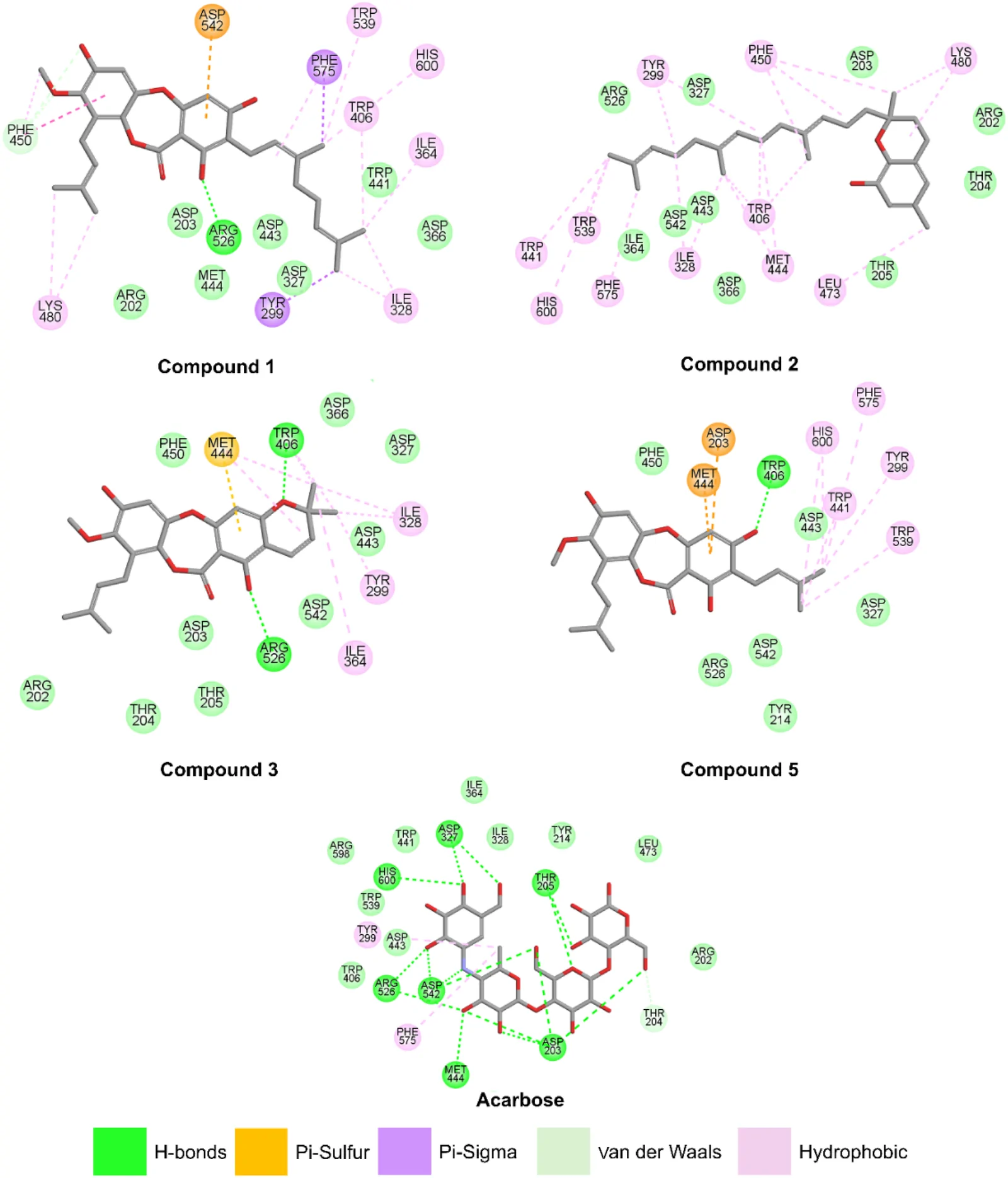
2D representations of ligand–protein interactions for selected compounds docked into the active site of Nt-MGAM, illustrating key residues involved in binding.

## Conclusions

In conclusion, the extraction and isolation of *G. parvifolia* fruits yielded two new depsidones, methyl garcidepsidone B (1) and parvifoliricodone A (6), together with known compounds, namely parvifoliol F (2), garcinisidone B (3), 5-acetoxy methyl furfural (4), and garcinisidone A (5) isolated from *G. parvifolia* fruits. The isolated compounds (1–6) of *G. parvifolia* fruits, showed antidiabetic, anti-tyrosinase, and antioxidant activities in the assays performed. Docking analysis identified interactions of bioactive compounds, such as methyl garcidepsidone B (1), which displayed the potent inhibitory activity with key binding site residues against α-glucosidase. Furthermore, these experimental results made the chemical basis for the benefits of *G. parvifolia* fruits towards free radical scavengers and controlling the blood glucose clear, and recommended further studies the isolated compounds from *G. parvifolia* fruits on mechanism of inhibition, selectivity studies, molecular dynamics simulations and binding free-energy calculations and *in vivo* assays.

## Author contributions

AZ and A-KN: conceptualization, data curation, formal analysis, investigation, methodology, validation, visualization, writing – original draft; JS, KU-H and DAL: data curation, investigation, methodology, writing – review & editing; ITK, YFHF, DNA and AAB performed the experiments; LR performed for provided taxonomy of plant; TR, SP and PP: conceptualization, methodology; RR: conceptualization, formal analysis, funding acquisition, project administration, resources, supervision, writing – original draft, writing – review & editing.

## Conflicts of interest

The authors declare no conflicts of interest.

## Supplementary Material

RA-016-D6RA06497C-s001

## Data Availability

The authors confirm that the data supporting the findings of this study are available within the article and its supplementary information (SI). Supplementary information: spectroscopic data, comprising 1D and 2D NMR spectra, together with method validation data and calibration curves. SI data for this article are available at the Dryad repository at: https://doi.org/10.5061/dryad.cnp5hqcng. See DOI: https://doi.org/10.1039/d6ra06497c.

## References

[cit1] Kardono L. B. S., Hanafi M., Sherly G., Kosela S., Harrison L. J. (2006). Pak. J. Biol. Sci..

[cit2] Santo B. L. S. E., Santana L. F., Junior W. H. K., de Araújo F. O., Bogo D., Freitas K. C., Guimarães R. C. A., Hiane P. A., Pott A., Filiú W. F. O., Asato M. A., Figueiredo P. O., Bastos P. R. H. O. (2020). Molecules.

[cit3] Shi F. Z., Fang Y. D., Fan M., Jiang X. J., Wang S., Wei G. Z. (2023). Fitoterapia.

[cit4] Trinh D. H., Huynh O. N., Nguyen T. Q., Nguyen H. T., Nguyen L. T., Pham H. D., Nguyen L. H. D., Tran P. T. (2021). Nat. Prod. Res..

[cit5] Sangeetha A., Fai C. W., Chan K. L., Yi M. V. J., Kooi M. S. (2021). J. Exp. Biol. Agric. Sci..

[cit6] Xu Y. J., Chiang P. Y., Lay Y. H., Vittal J. J., Wu X. H., Tan B. K. H., Imiyabir Z., Goh S. H. (2000). J. Nat. Prod..

[cit7] Zou D., Liu L., Liu F., Li D., Hua H. (2024). Chem. Biodiversity.

[cit8] Carillo-Hormaza L., Ramírez A. M., Quintero-Ortiz C., Cossio M., Medina S., Ferreres F., Gil-Izquierdo A., Osorio E. (2016). J. Funct. Foods.

[cit9] Gao X.-M., Cui D., Wang S.-S., Huang W.-Z., Li G.-P., Zheng G.-Z., Jiang M.-Y., Jiang Z.-Y. (2018). Phytochem. Lett..

[cit10] Peng X., Wang C., Hou Y., Tian J., Fan X., Li D., Hua H. (2023). Plants.

[cit11] Hassan S. H. A., Fry J. R., Bakar M. F. A. (2013). BioMed Res. Int..

[cit12] Shahid M., Fazry S., Azfaralariff A., Najm A. A. K., Law D., Mackeen M. M. (2023). Food Biosci..

[cit13] Chatatikum M., Supjaroen P., Promlat P., Chantarangkul C., Waranuntakul S., Nawarat J., Tangpong J., Chiabchalard A. (2020). Pharmacogn. J..

[cit14] Pailee P., Kuhakarn C., Sangsuwan C., Hongthong S., Piyachaturawat P., Suksen K., Jariyawat S., Akkarawongsapat R., Limthongkul J., Napaswad C., Kongsaeree P., Prabpai S., Jaipetch T., Pohmakotr M., Tuchinda P., Reutrakul V. (2018). Phytochemistry.

[cit15] Chantree P., Martviset P., Thongsepee N., Sangpairoi K., Sornchuer P. (2023). Nutrients.

[cit16] Choudhury B., Kandimalla R., Elancheran R., Bharali R., Kotoky J. (2018). Biomed. Pharmacother..

[cit17] Lakshmi C., Kumar K. A., Dennis T. J., Kumar T. S. S. P. N. S. S. (2011). Indian J. Pharm. Sci..

[cit18] Wang S.-N., Li Q., Jing M.-H., Alba E., Yang X.-H., Sabaté R., Han Y.-F., Pi R.-B., Lan W.-J., Yang X.-B., Chen J.-K. (2016). Neurochem. Res..

[cit19] Xu Y.-J., Chiang P.-Y., Lai Y.-H., Vittal J. J., Wu X.-H., Tan B. K. H., Imiyabir Z., Goh S.-H. (2000). J. Nat. Prod..

[cit20] Rukachaisirikul V., Naklue W., Phongpaichit S., Towatana N. H., Maneenoon K. (2006). Tetrahedron.

[cit21] Ito C., Itoigawa M., Mishina Y., Tomiyasu H., Litaudon M., Cosson J.-P., Mukainaka T., Tokuda H., Nishino H., Furukawa H. (2001). J. Nat. Prod..

[cit22] Kim H.-Y., Park S.-H., Zuo G., Kim K. H., Hwang S. H., Suh H.-W., Lim S.-S. (2021). Molecules.

[cit23] Ito C., Miyamoto Y., Nakayama M., Kawai Y., Rao K. S., Furukawa H. (1997). Chem. Pharm. Bull..

[cit24] Ramadhan R., Phuwapraisirisan P. (2015). Bioorg. Med. Chem. Lett..

[cit25] Khongkarat P., Ramadhan R., Phuwapraisirisan P., Chanchao C. (2020). Heliyon.

[cit26] Khongkarat P., Ramadhan R., Phuwapraisirisan P., Chanchao C. (2021). J. Apic. Sci..

[cit27] Pawlowicz K., Paczkowska-Walendowska M., Osmalek T., Cielecka-Piontek J. (2022). Pharmaceutics.

[cit28] Wairata J., Fadlan A., Purnomo A. S., Taher M., Ersam T. (2022). Arabian J. Chem..

[cit29] Jones G., Willett P., Glen R. C., Leach A. R., Taylor R. (1997). J. Mol. Biol..

[cit30] Todsaporn D., Sanachai K., Aonbangkhen C., Geronikaki A., Kartsev V., Lichitsky B., Komogortsev A., Maitarad P., Rungrotmongkol T. (2025). Comput. Struct. Biotechnol. J..

[cit31] Sim L., Jayakanthan K., Mohan S., Nasi R., Johnston B. D., Pinto B. M., Rose D. R. (2010). Biochemistry.

[cit32] Meng E. C., Goddard T. D., Pettersen E. F., Couch G. S., Pearson Z. J., Morris J. H., Ferrin T. E. (2023). Protein Sci..

[cit33] Ha L. D., Hansen P. E., Duus F., Pham H. D., Nguyen L. H. D. (2012). Magn. Reson. Chem..

[cit34] Permana D., Lajis N. H., Mackeen M. M., Ali A. M., Aimi N., Kitajima M., Takayama H. (2001). J. Nat. Prod..

[cit35] Kosela S., Cao S. G., Wu X. H., Vittal J. J., Sukri T., Masdianto, Goh S. H., Sim K. Y. (1999). Tetrahedron Lett..

[cit36] Tran Q. H. N., Nguyen H. T. M., Nguyen T. H., Nguyen T. T. L., Sichaem J., Do L. T. M. (2025). Nat. Prod. Res..

[cit37] Thu H. N. T., Minh Q. P., Van C. P., Van T. N., Van K. P., Thanh T. N., Tu A. L. T., Litaudon M., The S. N. (2021). Chem. Biodiversity.

[cit38] Platzer M., Kiese S., Herfellner T., Schweiggert-Weisz U., Miesbauer O., Eisner P. (2021). Molecules.

[cit39] Althaher A. R., Mastinu A. (2023). Appl. Sci..

[cit40] Bay M. V., Nam P. C., Quang D. T., Mechler A., Hien N. K., Hoa N. T., Vo Q. V. (2020). ACS Omega.

[cit41] Fernandes R. P. P., Trindade M. A., Tonin F. G., Lima C. G., Pugine S. M. P., Munekata P. E. S., Lorenzo J. M., de Melo M. P. (2016). J. Food Sci. Technol..

