# Supplementary material for: New depsidones from Garcinia parvifolia and their antidiabetic, anti-tyrosinase, and antioxidant activities
Source: RSC Adv. 2026 Oct 22;16(48):65262–70. doi: 10.1039/d6ra06497c (PMC13688104; doi:10.1039/d6ra06497c)
Supplement: Supplementary file 1 [file RA-016-D6RA06497C-s001.pdf]

Supplementary Information (SI) for RSC Advances.
This journal is © The Royal Society of Chemistry

Supplementary data

New Depsidones from *Garcinia parvifolia* and their Antidiabetic, Anti-tyrosinase, and Antioxidant Activities

Rico Ramadhan[a,b,c*], Azeana Zahari[d], Irmayanti Tri Kurnia[a], Anh-Khoa Nguyen[e], Sehanat Prasongsuk[c], Yazdaniyar Fajri Halimi Firdaus[f], Thanyada Rungrotmongkol[f,g], Preecha Phuwapraisirisan[h], Dinda Ayu Lestari[a], Jirapast Sichaem[i], Kautsar Ul-Haq[a,b], Aziz Ahmad Bukhori[b], Devi Nawang Ariningtyas[b], Lukman Riyadi[j]

a. Division of Exploration and Synthesis of Bioactive Compounds (ESBC), University CoE – Research Center for Bio-Molecule Engineering (BIOME), Universitas Airlangga, Surabaya 60115, Indonesia.
b. Department of Chemistry, Faculty of Science and Technology, Universitas Airlangga, Surabaya 60115, Indonesia
c. Plant Biomass Utilization Research Unit, Department of Botany, Faculty of Science, Chulalongkorn University, Bangkok 10330, Thailand
d. Department of Chemistry, Faculty of Science, Universiti Malaya, Kuala Lumpur 50603, Malaysia
e. Faculty of Pharmacy, Ton Duc Thang University, Ho Chi Minh City 758307, Vietnam
f. Center of Excellence in Biocatalyst and Sustainable Biotechnology, Department of Biochemistry, Faculty of Science, Chulalongkorn University, Bangkok 10330, Thailand
g. Program in Bioinformatics and Computational Biology, College of Interdisciplinary and Integrative Studies, Chulalongkorn University, Bangkok 10330, Thailand.
h. Center of Excellent in Natural Products (CENP), Department of Chemistry, Faculty of Science, Chulalongkorn University, Bangkok 10330, Thailand.
i. Department of Chemistry, Faculty of Science and Technology, Thammasat University Lampang Campus, Lampang 52190, Thailand.
j. Environmental Office of Balikpapan, Balikpapan 76115, East Kalimantan, Indonesia.

*Corresponding author:

Rico Ramadhan

Address: Kampus Merr C Mulyorejo, Faculty of Science and Technology, Universitas Airlangga, Surabaya, East Java, Indonesia, 60115

E-mail: rico.ramadhan@fst.unair.ac.id

Phone/Fax: +6231-5922427

NMR spectrum of isolated compounds

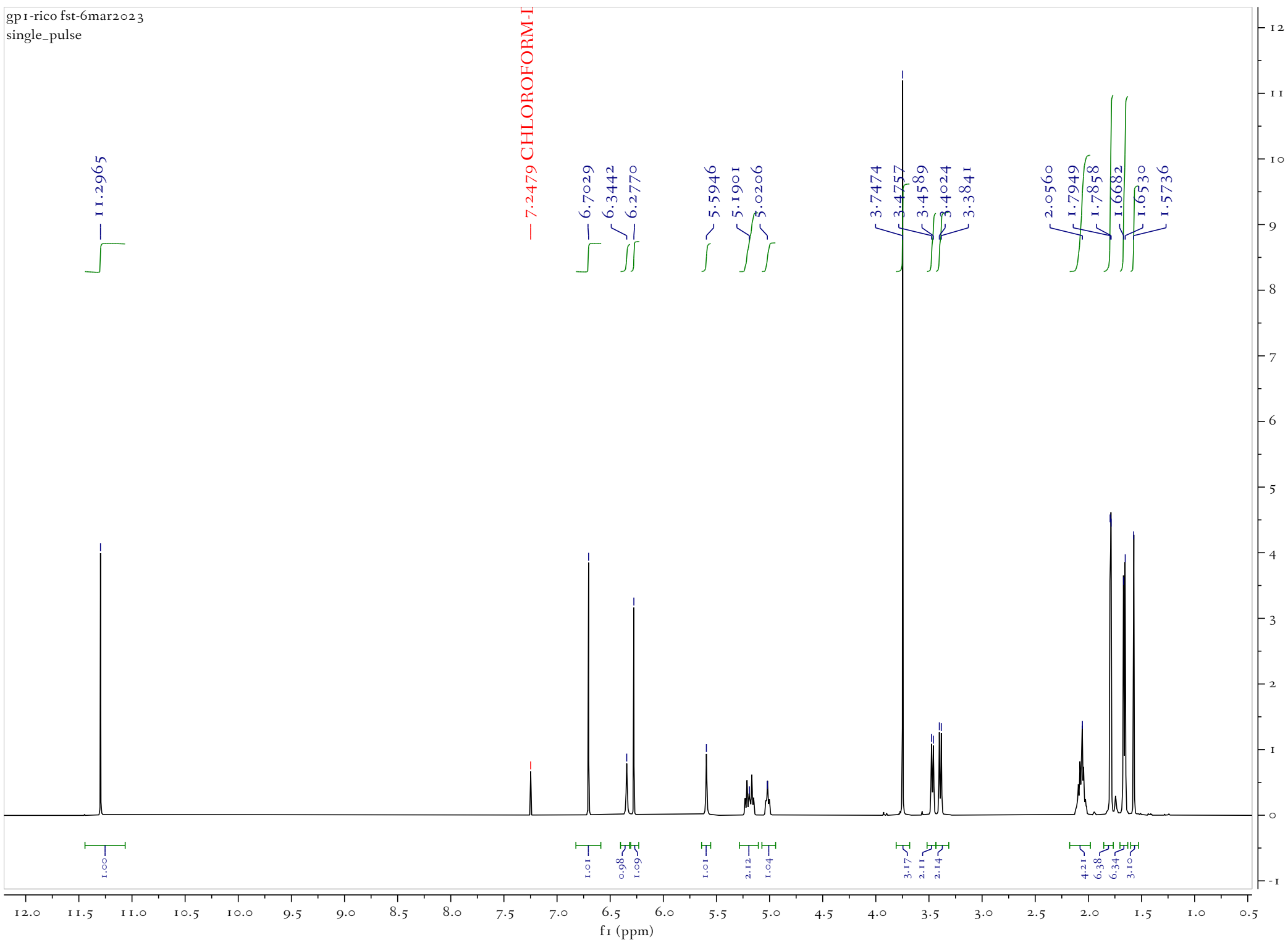

Figure S1. ^{1}H NMR spectrum of **1** ($CDCl_3$)

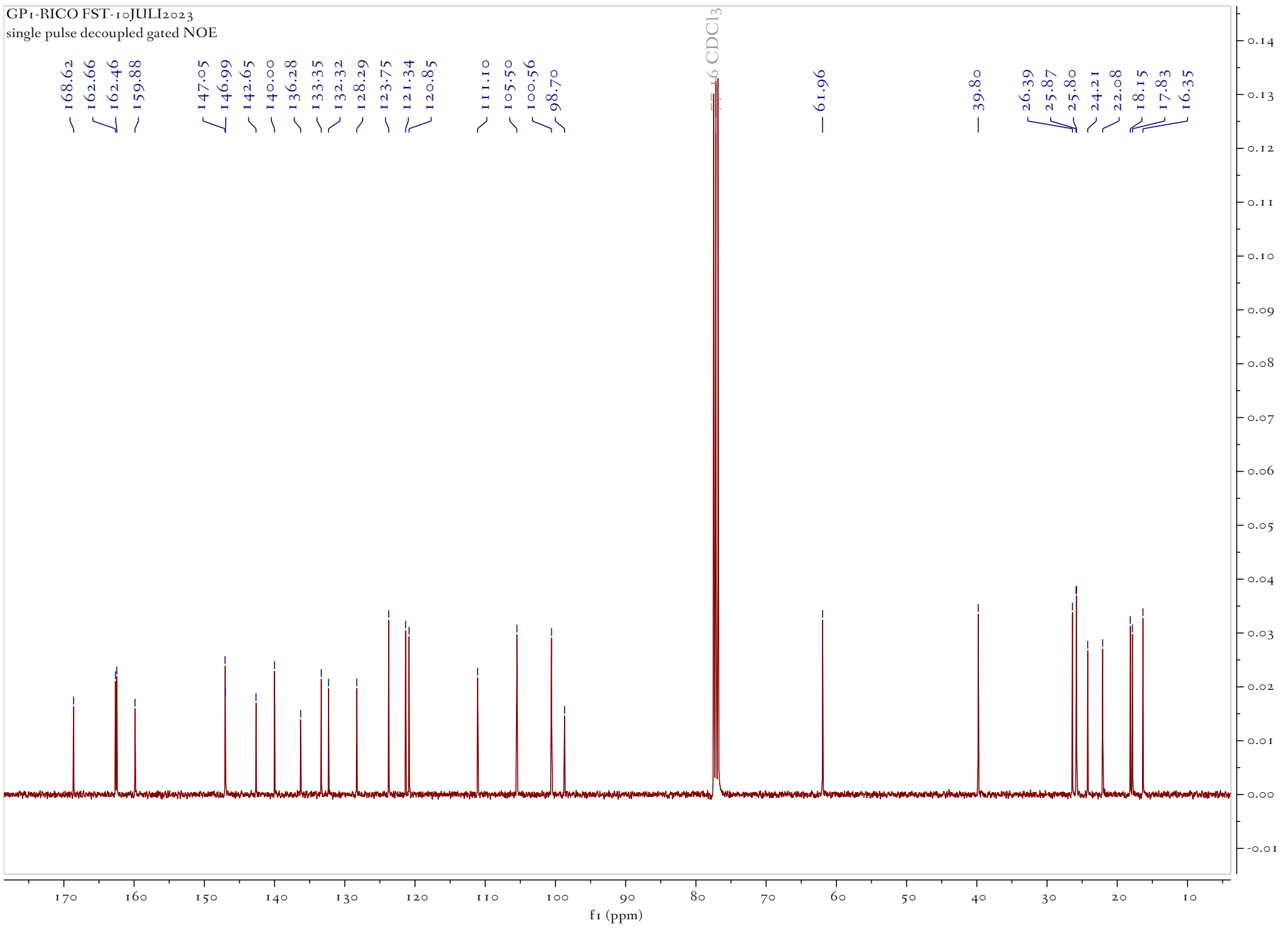

Figure S2. ^{13}C NMR spectrum of **1** ($CDCl_3$)

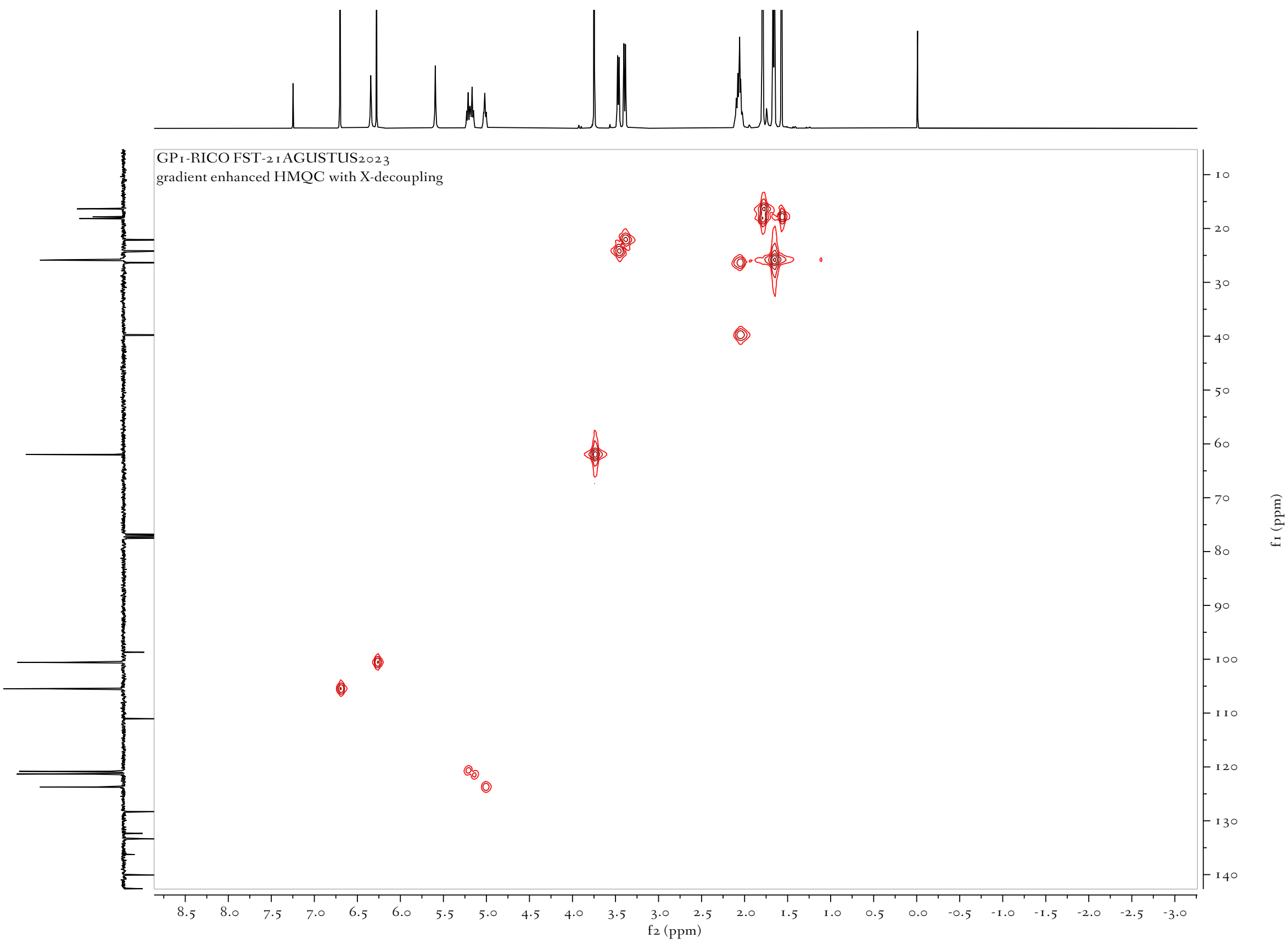

Figure S3. HMQC spectrum of **1** ($CDCl_3$)

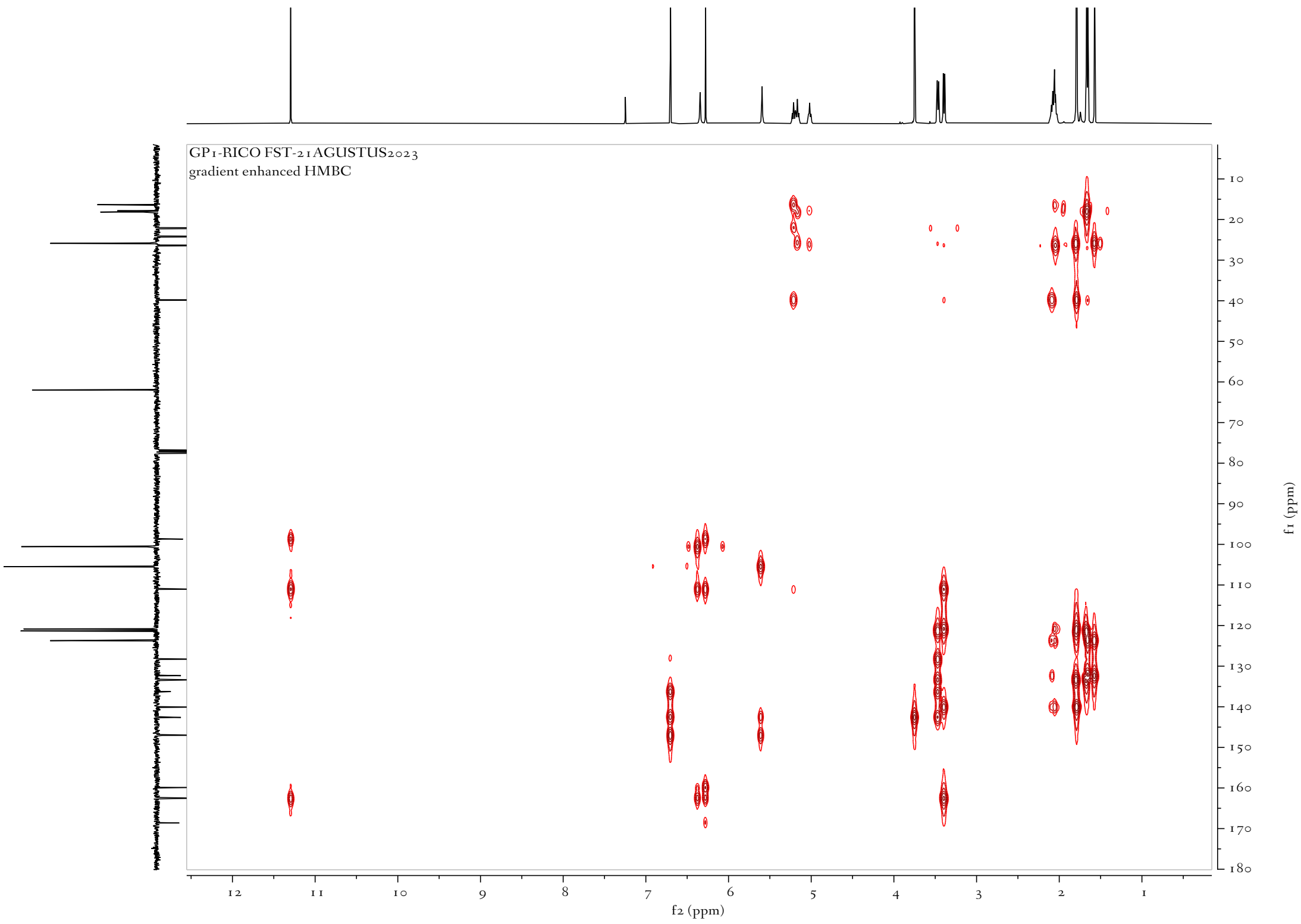

Figure S4. HMBC spectrum of **1** ($CDCl_3$)

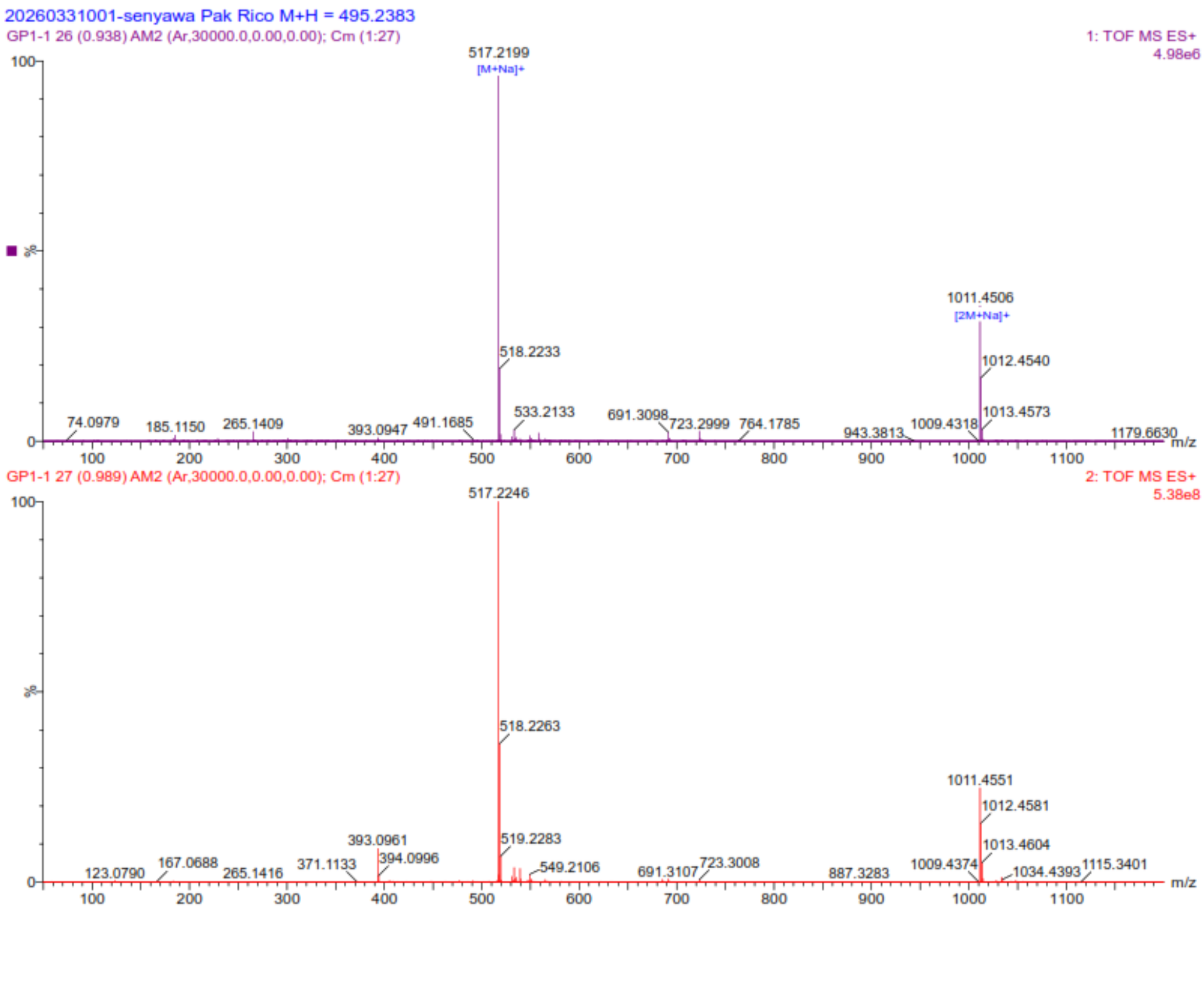

Figure S5. HRMS spectrum of **1**.

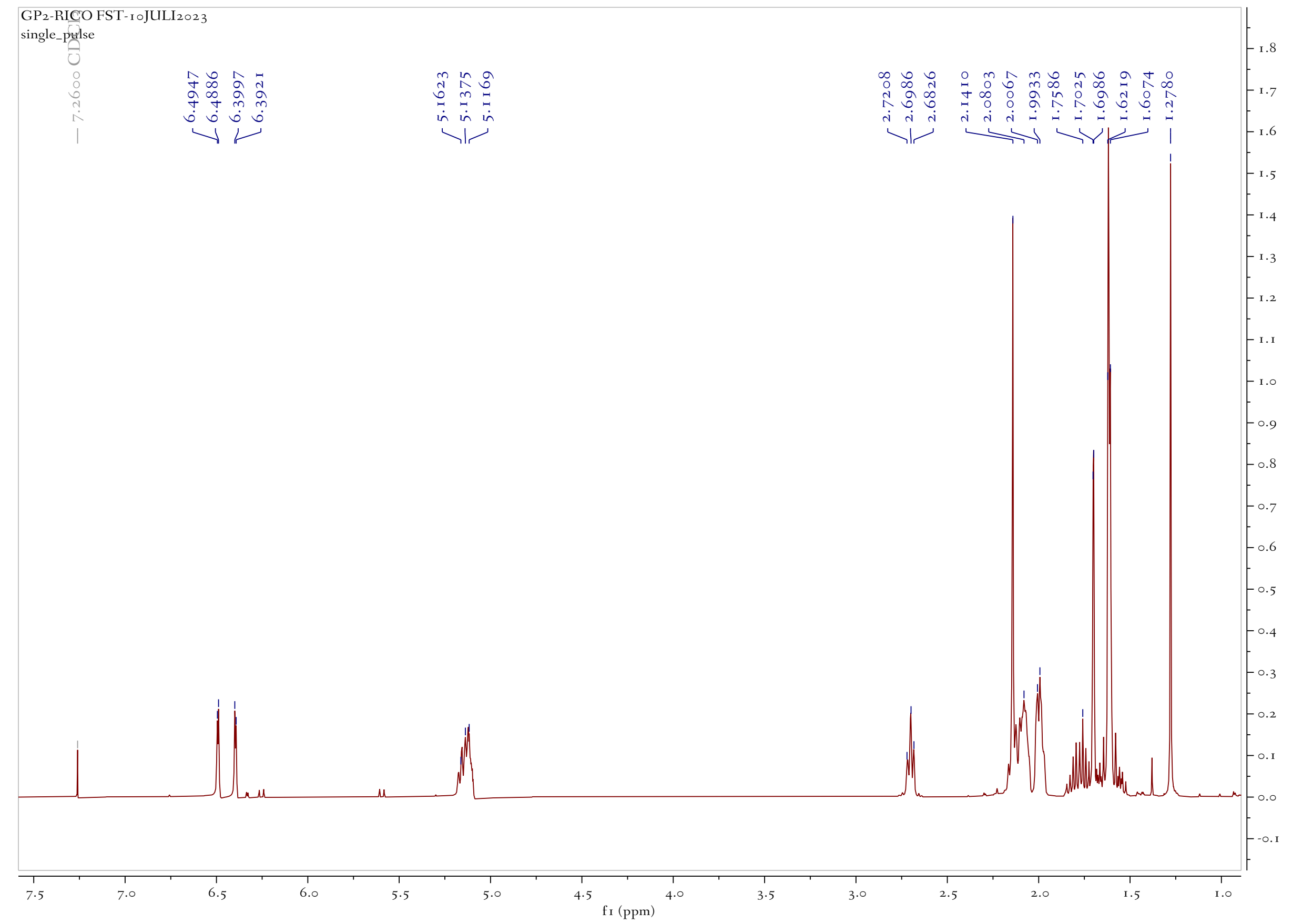

Figure S6. ^{1}H NMR spectrum of **2** ($CDCl_3$)

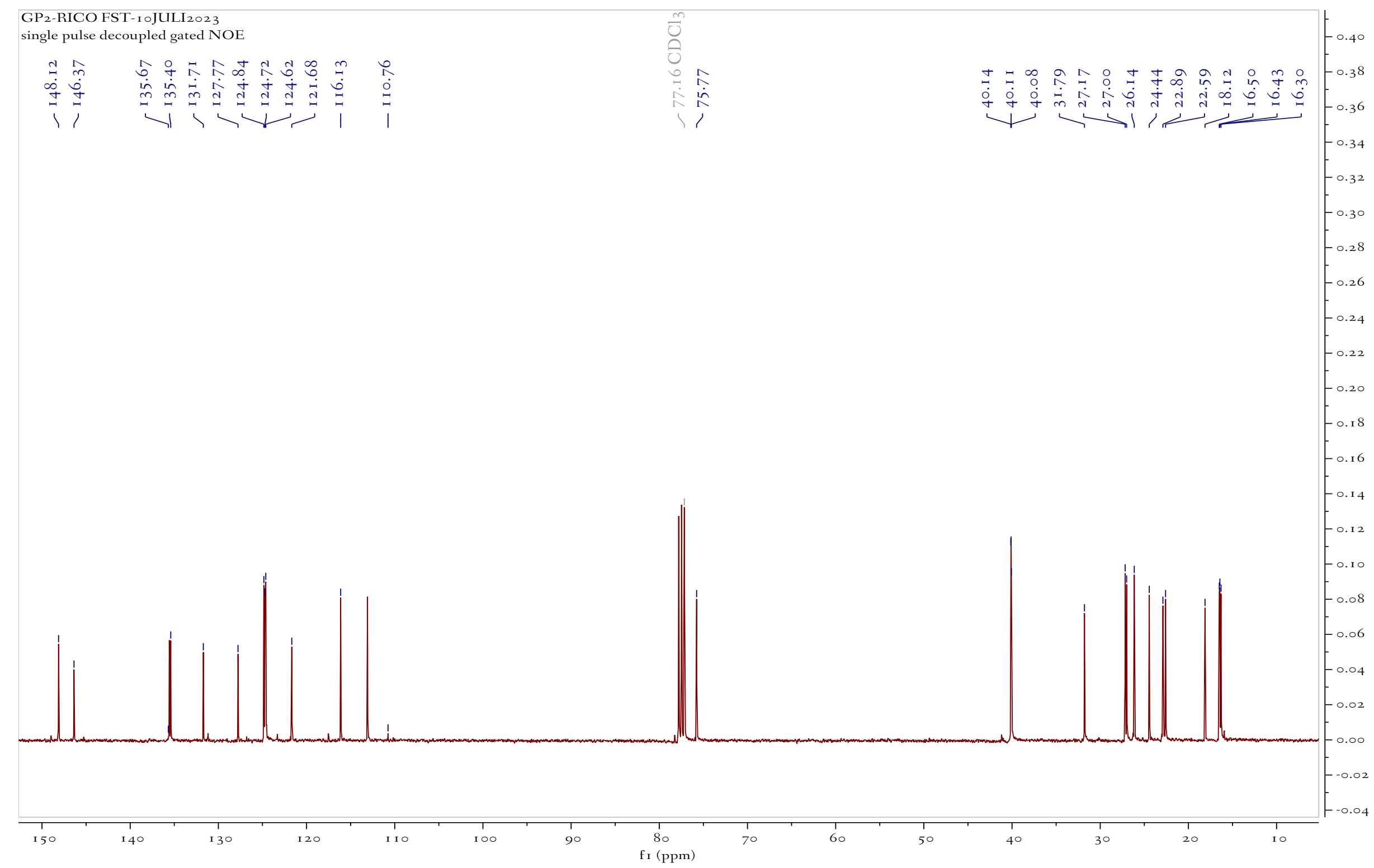

Figure S7. ^{13}C NMR spectrum of **2** ($CDCl_3$)

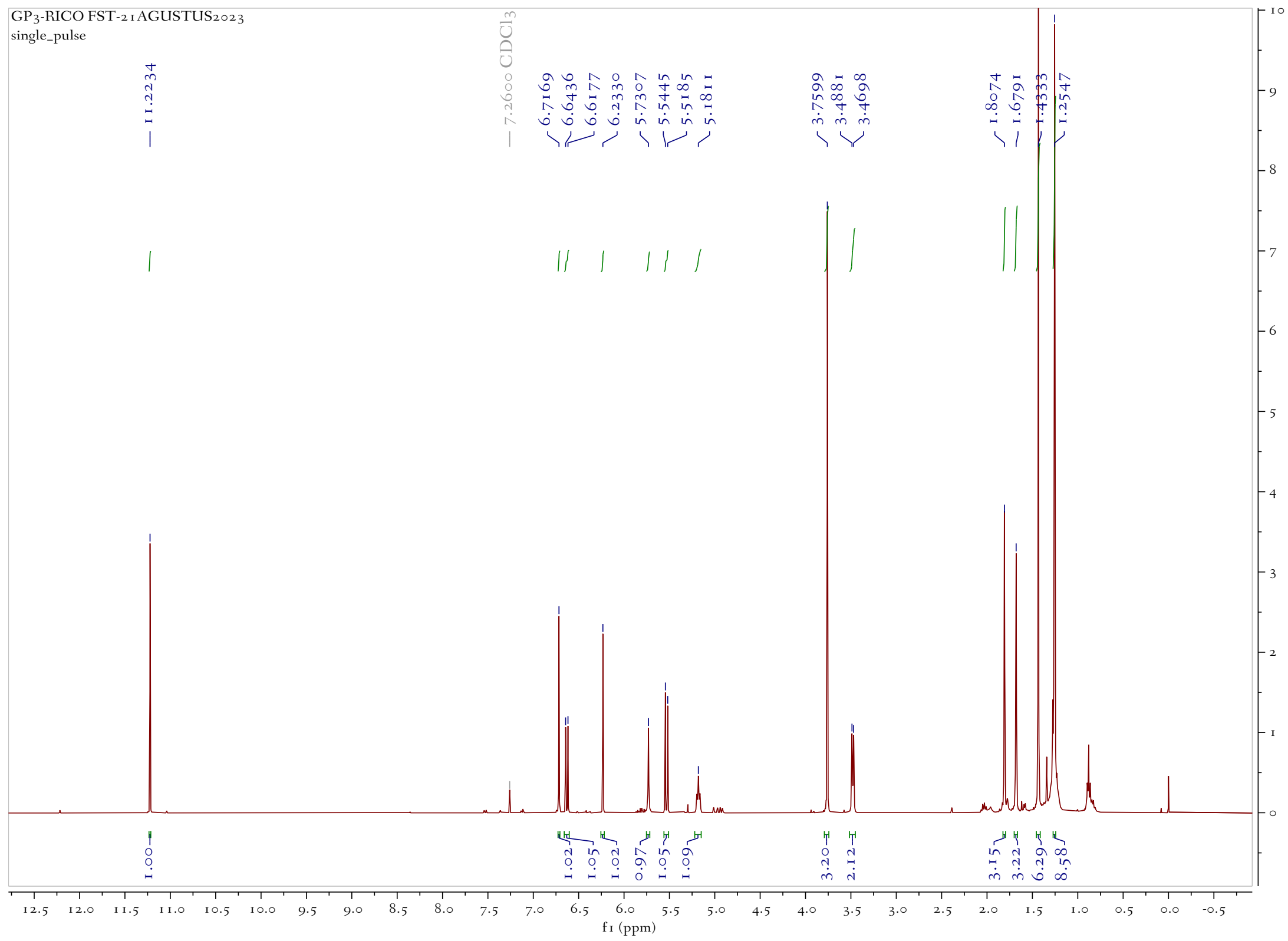

Figure S8. ^{1}H NMR spectrum of **3** ($CDCl_3$)

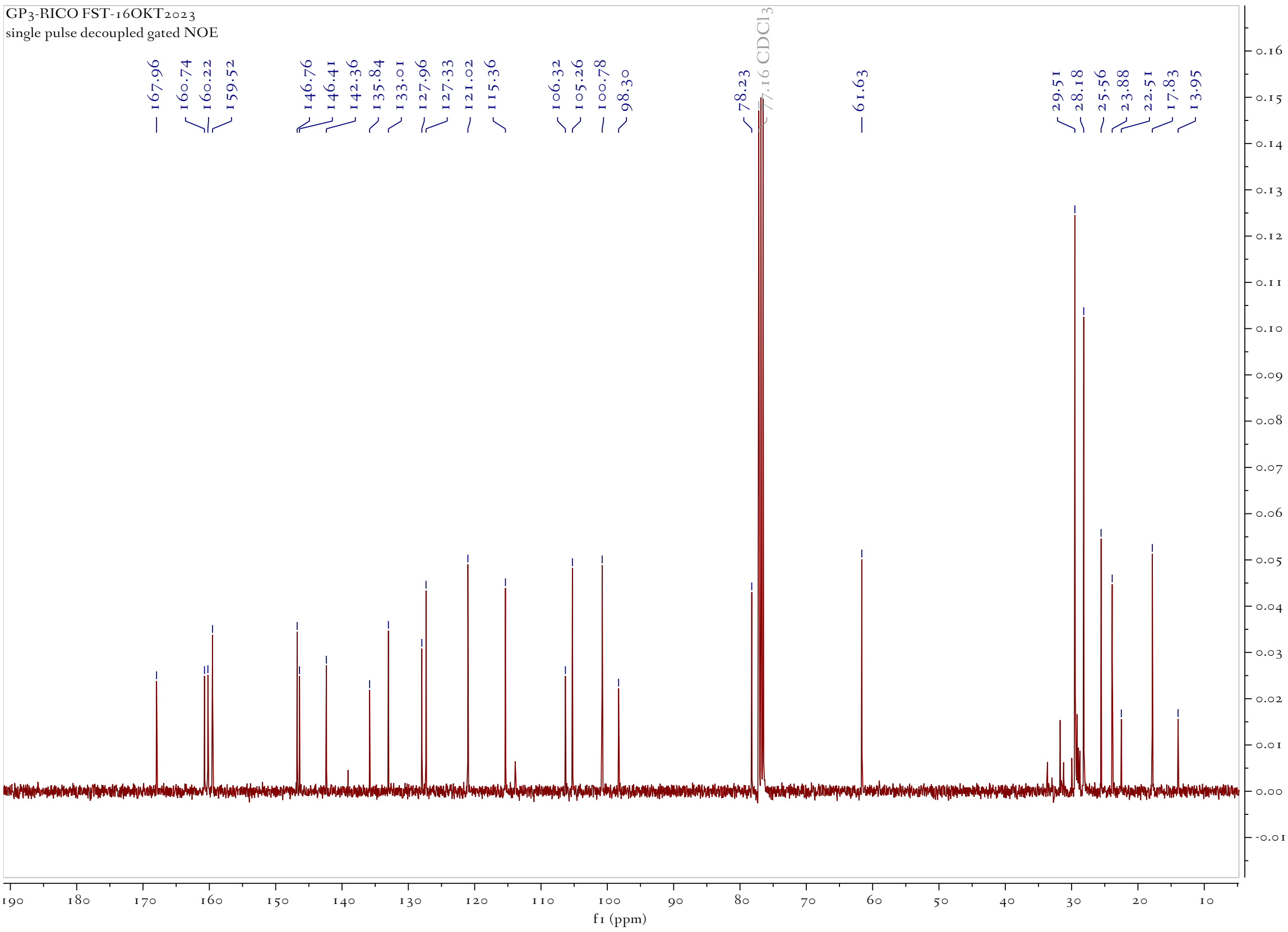

Figure S9. ^{13}C NMR spectrum of **3** ($CDCl_3$)

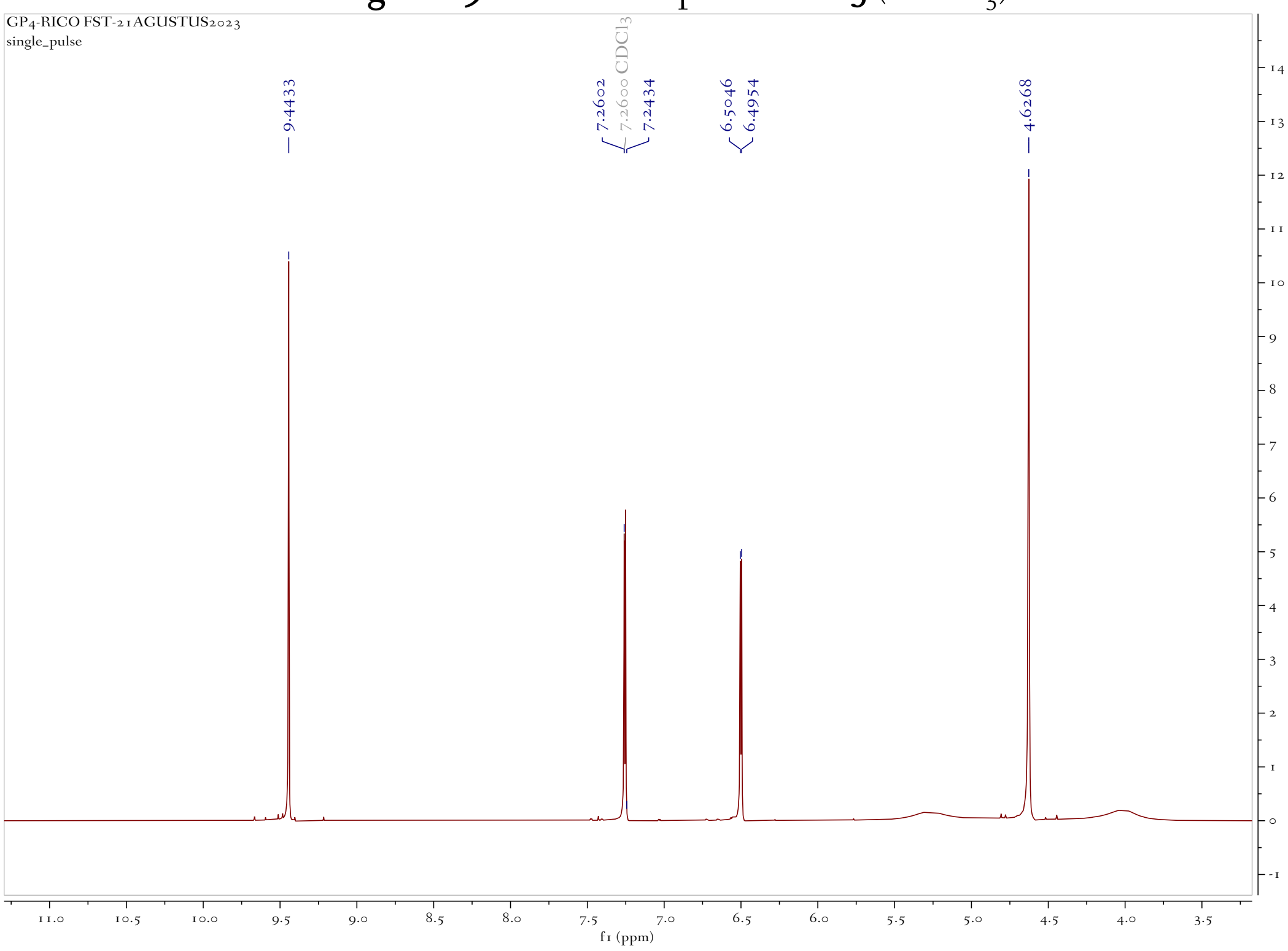

Figure S10. ^{1}H NMR spectrum of **4** ($CDCl_3$)

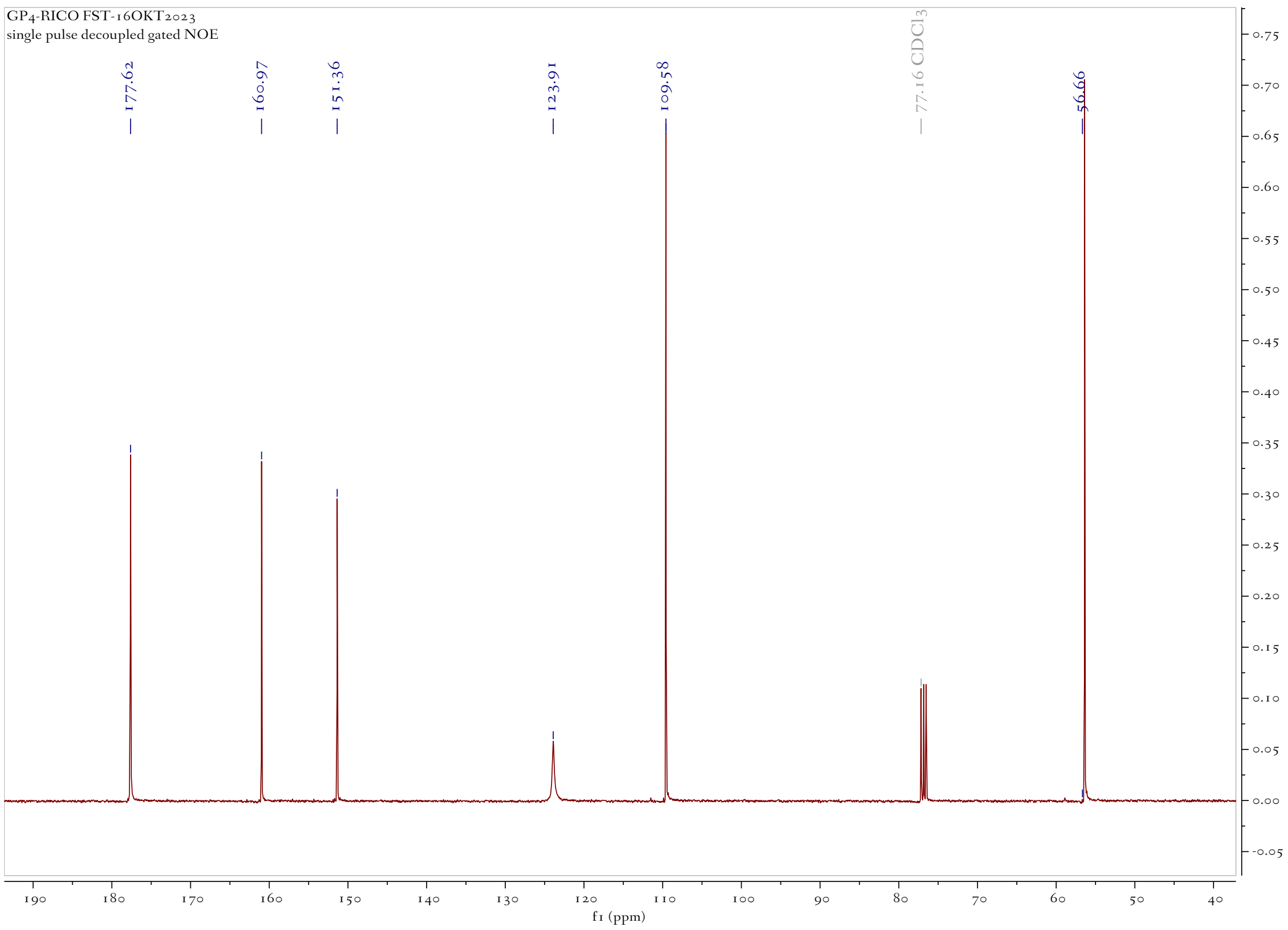

Figure S11. ^{13}C NMR spectrum of **4** ($CDCl_3$)

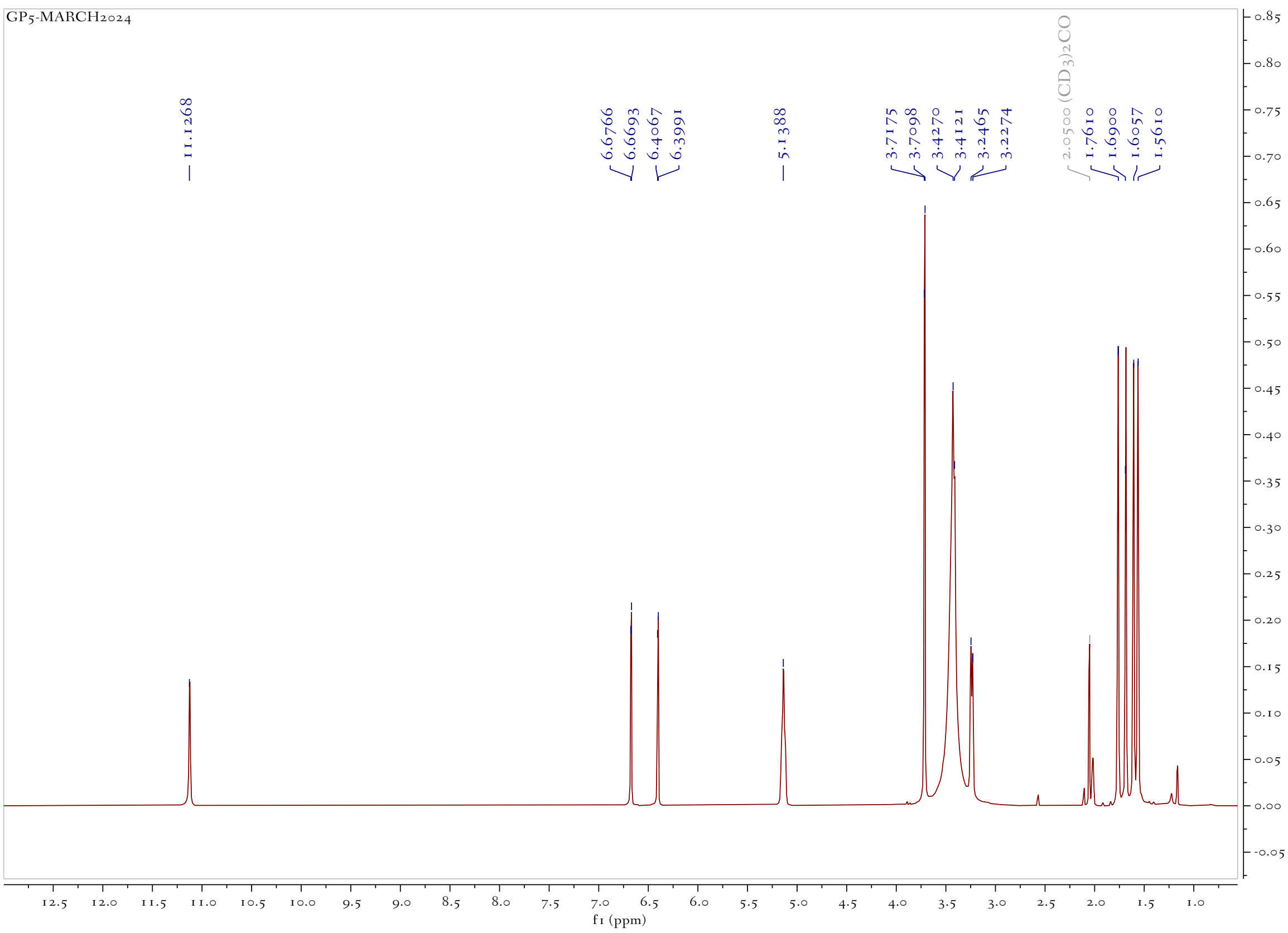

Figure S12. ^{1}H NMR spectrum of **5** (Acetone-d6)

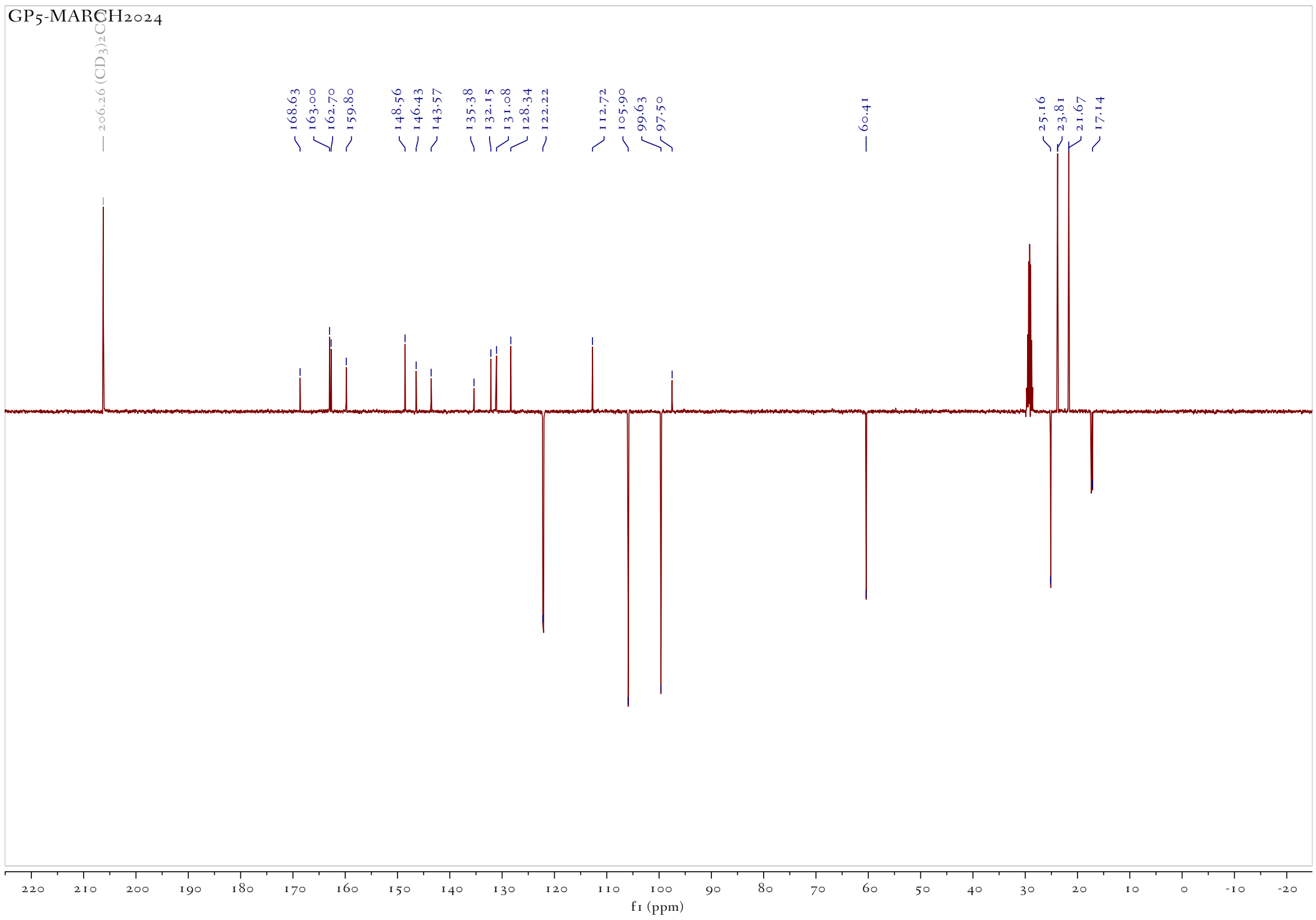

Figure S12. ^{13}C-APT spectrum of **5** (Acetone-d6)

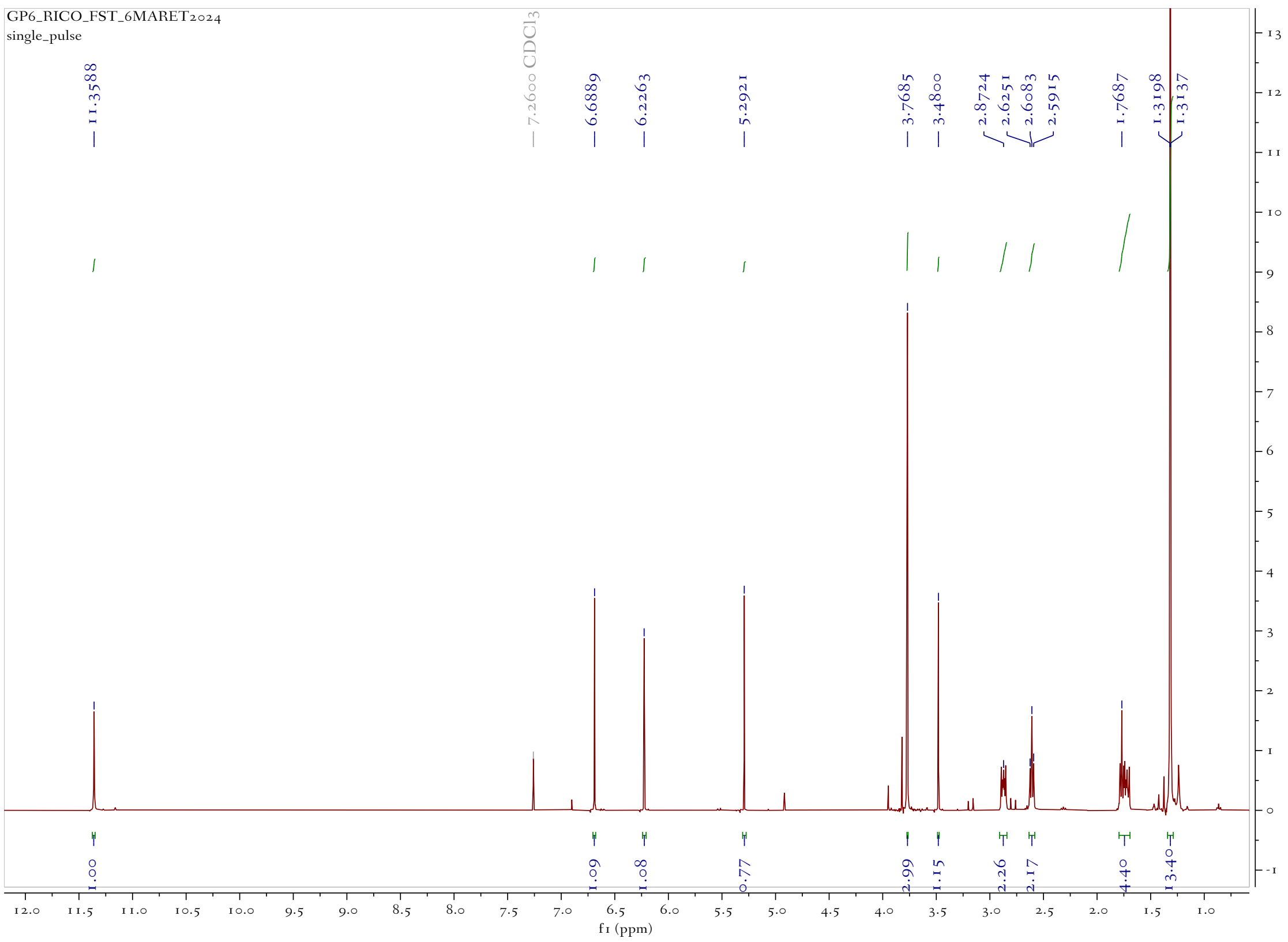

Figure S13. ^{1}H spectrum of **6** ($CDCl_3$)

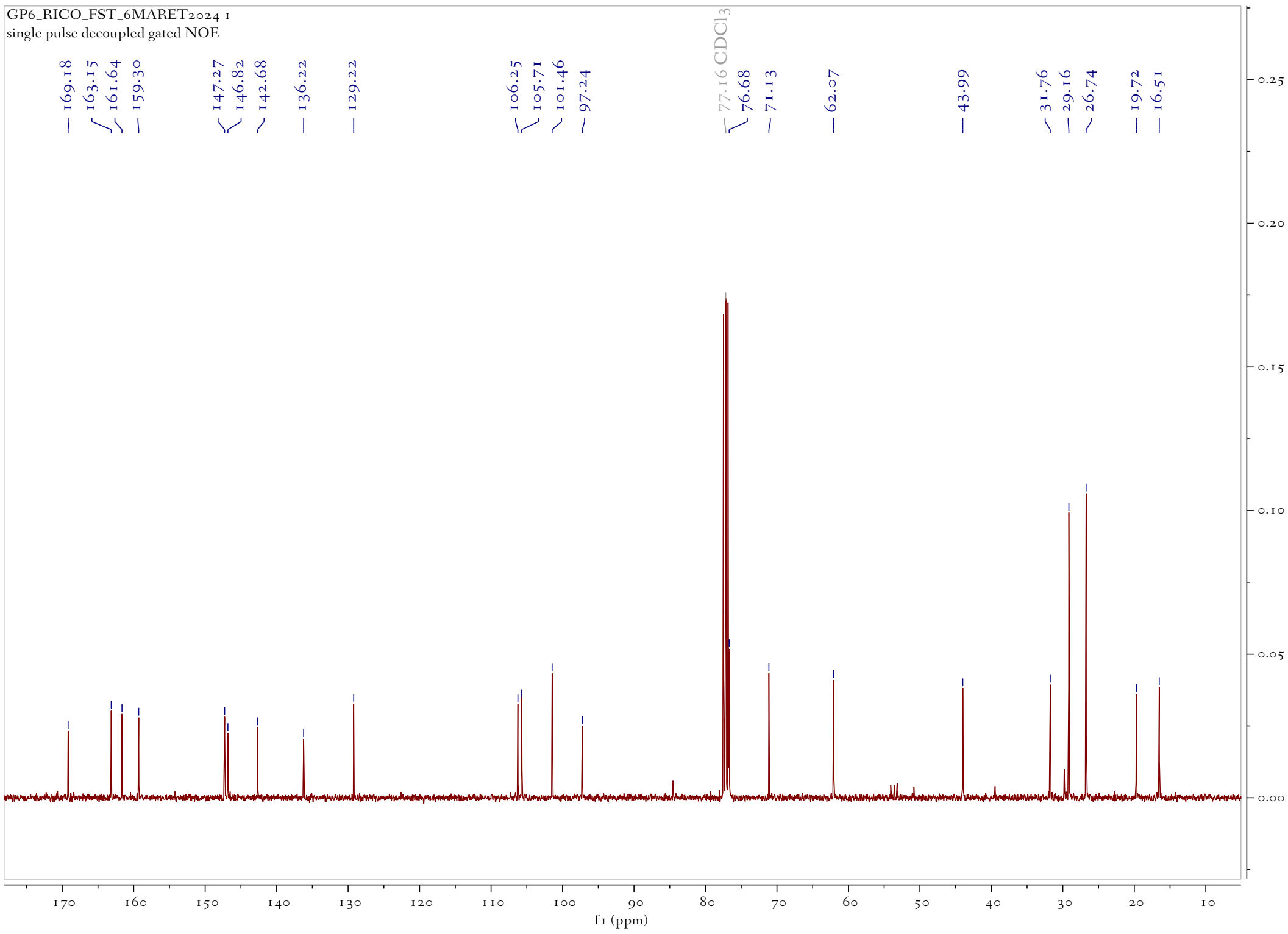

Figure S14. ^{13}C spectrum of **6** ($CDCl_3$)

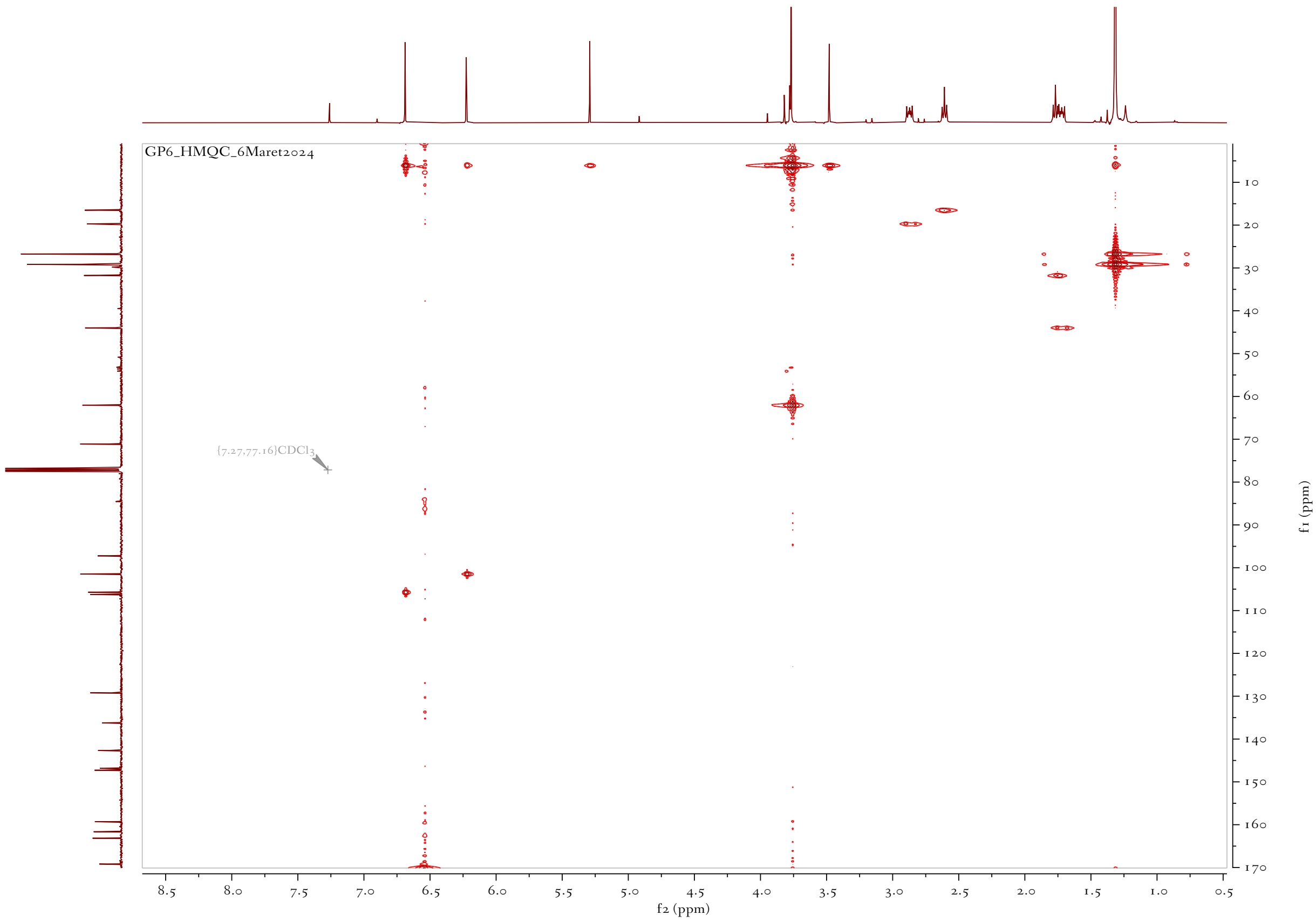

Figure S15. HMQC spectrum of **6** ($CDCl_3$)

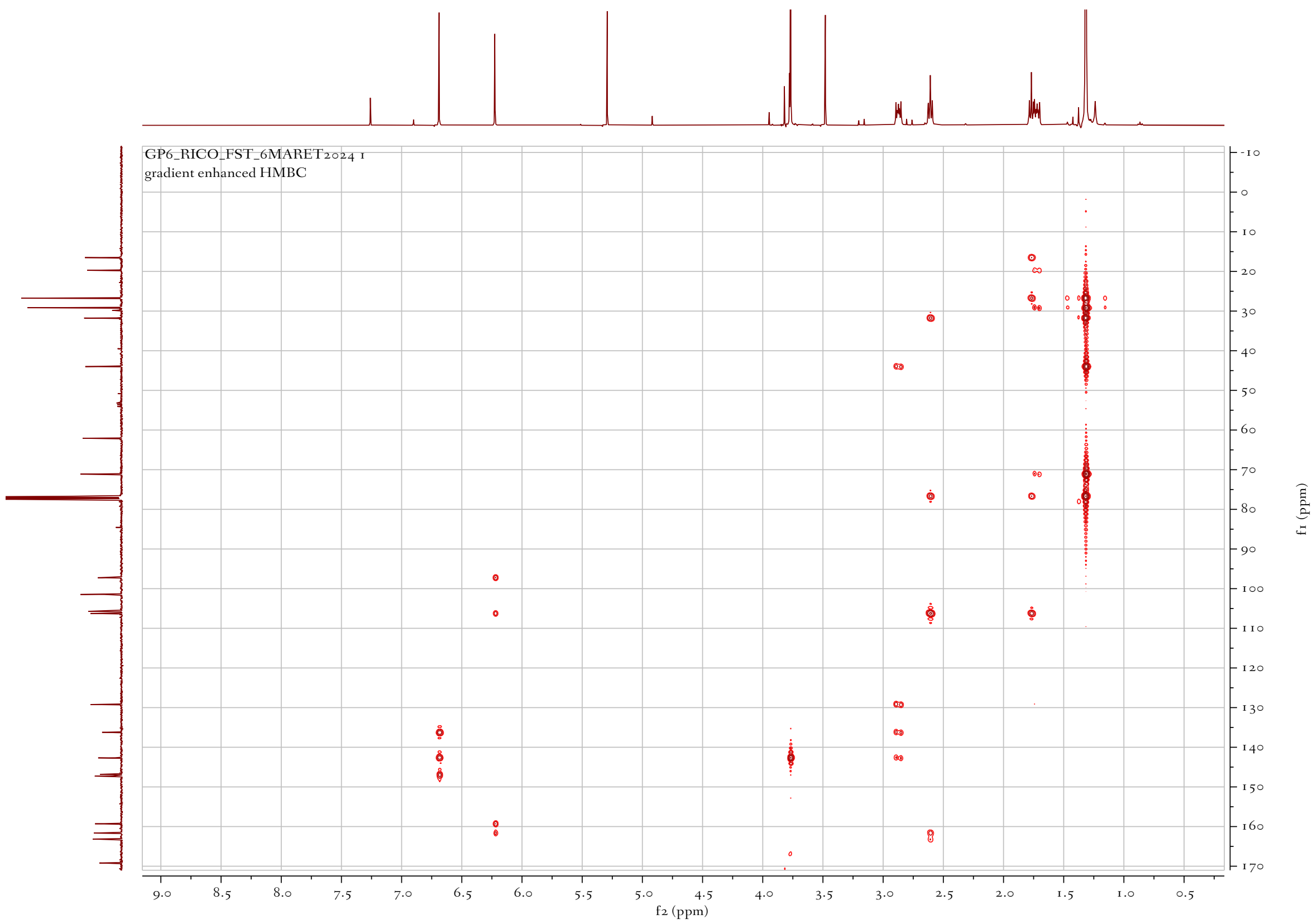

Figure S16. HMBC spectrum of **6** ($CDCl_3$)

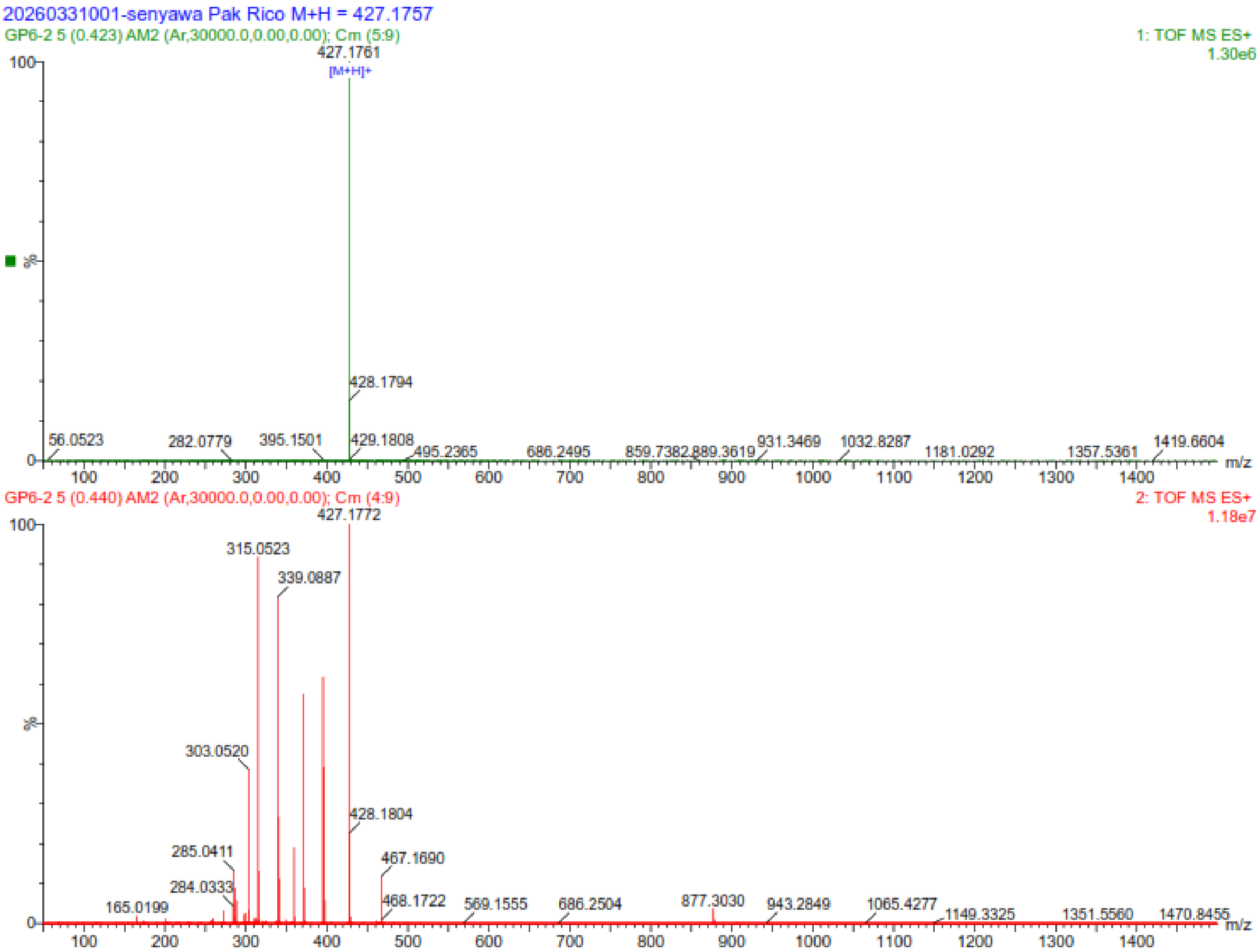

Figure S16. HRMS spectrum of **6** ($CDCl_3$)

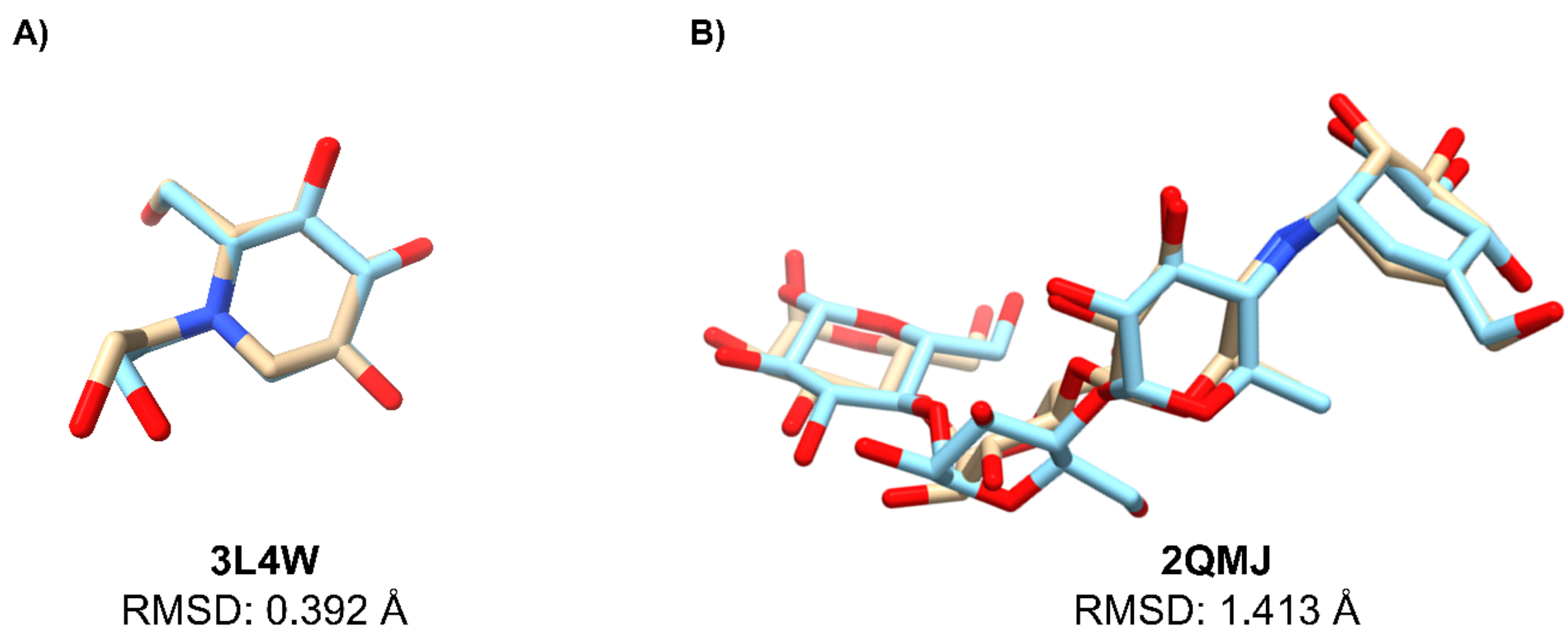

Figure S17. Validation of the docking protocol. **(A)** Re-docking of the co-crystallized ligand miglitol into the active site of Nt-MGAM (3L4W), demonstrating close correspondence between the docked and crystallographic binding conformations. **(B)** Superimposition of the docked acarbose in Nt-MGAM (PDB ID: 3L4W) with the crystallographic pose of acarbose from PDB ID: 2QMJ, yielding an RMSD of 1.413 Å, indicating a similar binding orientation within the catalytic pocket.

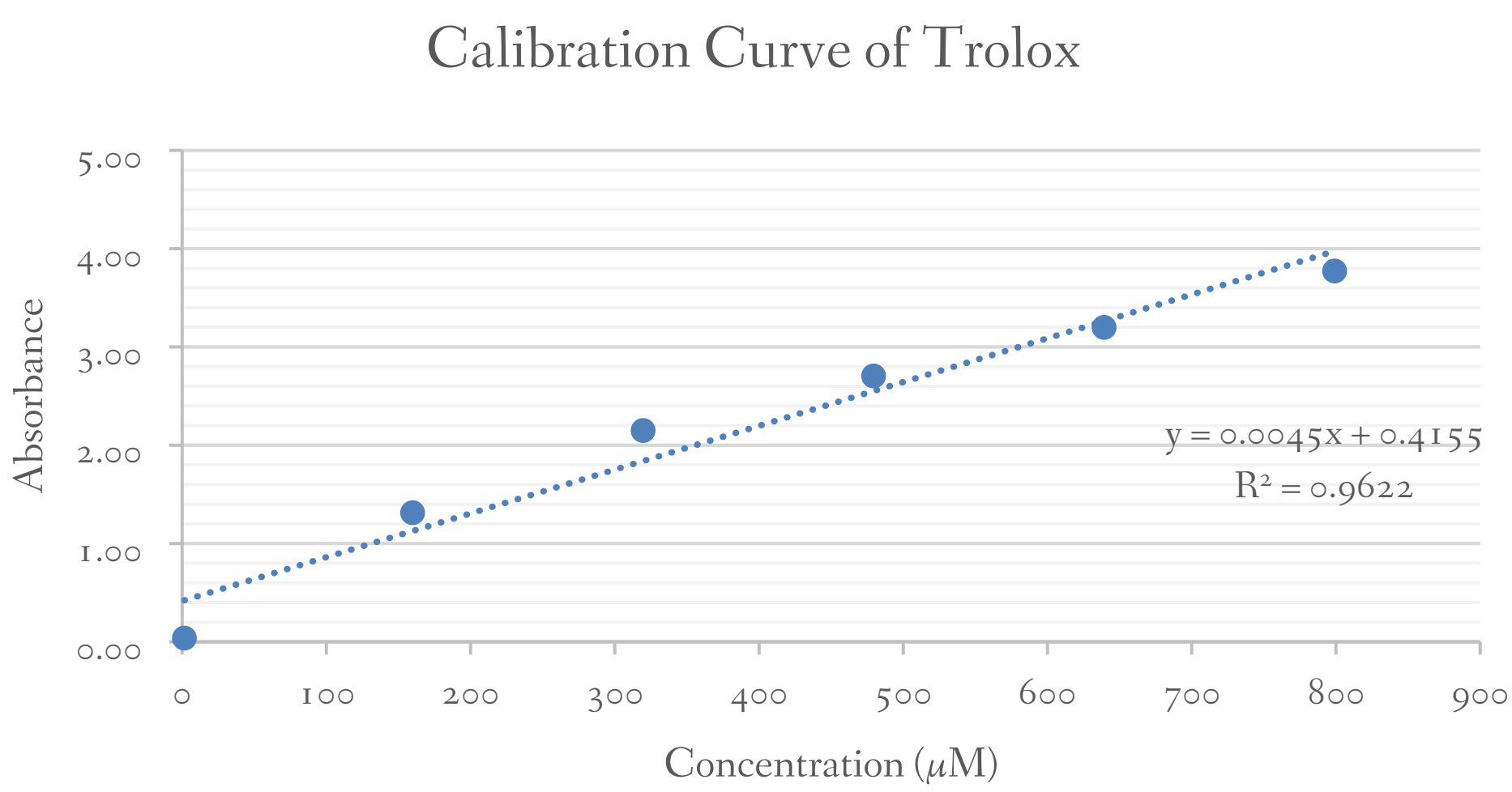

Figure S18. Calibration curve of CUPRAC

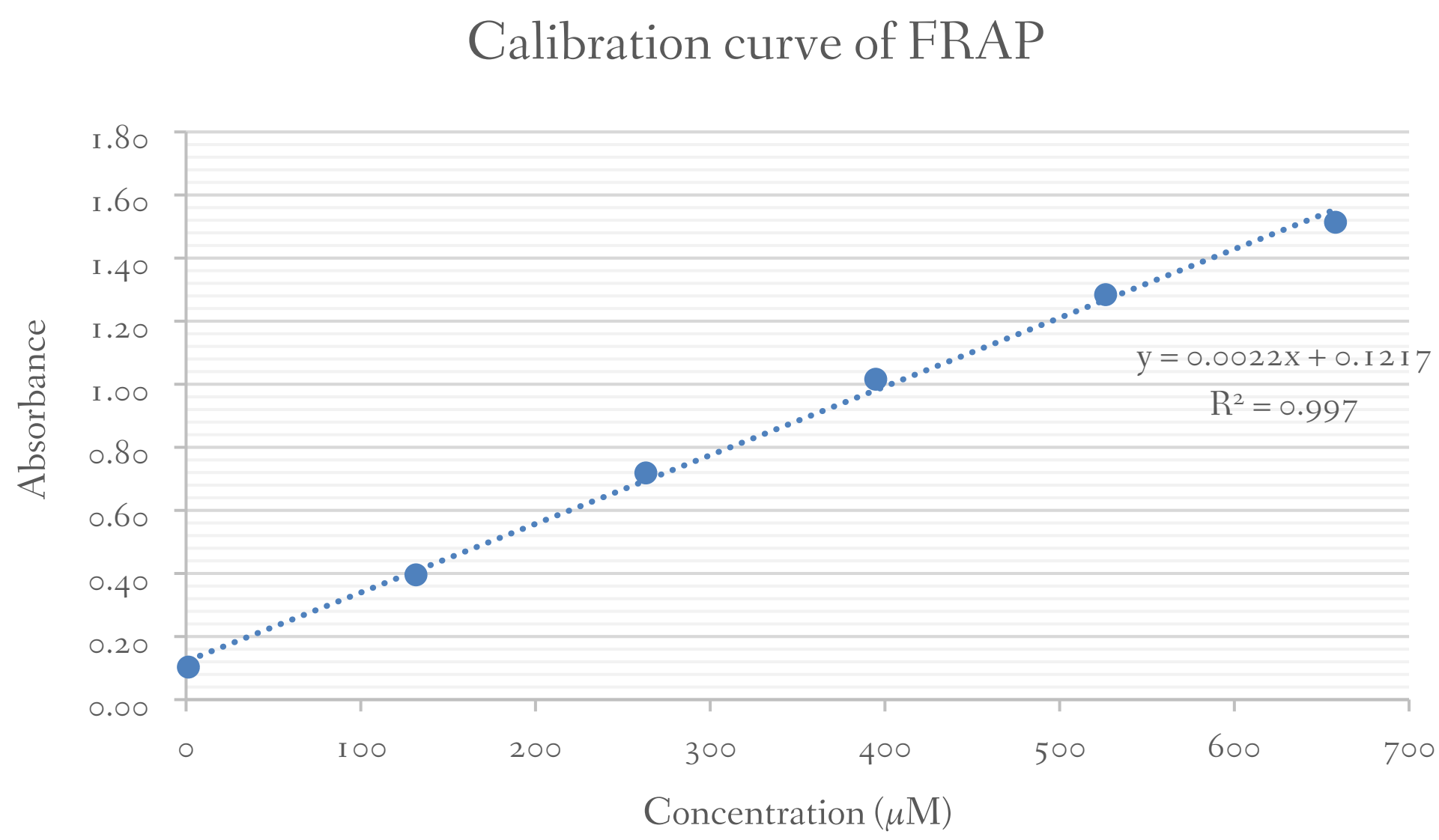

Figure S19. Calibration curve of FRAP

Note: The calculations for CUPRAC and FRAP are also supported by the references provided below:

1. R. Apak, K Güçlü, M. Özyürek, S. E. Karademir, E. Erçağ, *Int J Food Sci Nutr*, 2006, 57, 292-304, Doi: 10.1080/09637480600798132.
2. J. S. Soun, Y. J. Kim, H. J. Na, H. R. Jung, C. K. Song, S. Y. Kang, J. Y. Kim, Food Sci Biotechnol, 2019, 28, 201-207, Doi: 10.1007/s10068-018-0352-y.

Table S1. GOLD scores were correlated with pIC_{50} values for the five compounds

Compound	IC_{50} (µM)	Potency rank	GOLD Score	Binding Rank	Spearman's ρ	Pearson coefficient (r)
1	14.87	1	67.20	1	r = 0.70, p =0.188	r = 0.639 (r^2 = 0.41), p = 0.246
2	18.97	2	63.03	3		
3	25.61	4	65.15	2		
5	21.15	3	57.75	4		
6	26.93	5	52.42	5		
